# Dorsolateral and medial prefrontal cortex mediate the influence of incidental priming on economic decision making in obesity

**DOI:** 10.1038/s41598-018-35834-1

**Published:** 2018-12-04

**Authors:** Filip Morys, Stefan Bode, Annette Horstmann

**Affiliations:** 1 Leipzig University Medical Centre, IFB Adiposity Diseases, 04103 Leipzig, Germany; 2 Department of Neurology, Max Planck Institute for Human Cognitive and Brain Sciences, 04103 Leipzig, Germany; 3 The University of Melbourne, Melbourne School of Psychological Sciences, Parkville, VIC 3010 Australia; 4 Department of Psychology, University of Cologne, 50969 Cologne, Germany; 5 Leipzig University Medical Centre, Collaborative Research Centre 1052-A5, 04103 Leipzig, Germany

## Abstract

Obese individuals discount future rewards to a higher degree than lean individuals, which is generally considered disadvantageous. Moreover, their decisions are altered more easily by decision-irrelevant cues. Here, we investigated neural correlates of this phenomenon using functional MRI. We tested 30 lean and 26 obese human subjects on a primed delay discounting paradigm using gustatory and visual cues of positive, neutral and negative valence to bias their intertemporal preferences. We hypothesised that activation differences in reward-related and behavioural control areas, and changes in connectivity between these areas, would reflect the effect of these cues. Here, obese subjects were more susceptible to priming with negative gustatory cues towards delayed choices as opposed to lean subjects. This was related to lower activity in the left dorsolateral prefrontal cortex during priming. Modulation of functional connectivity between the dlPFC and the ventromedial PFC by the behavioural priming effect correlated negatively with BMI. This might indicate that default goals of obese individuals were different from those of lean participants, as the dlPFC has been suggested to be involved in internal goal pursuit. The present results further our understanding of the role of the PFC in decision-making and might inform future weight-management approaches based on non-invasive brain stimulation.

## Introduction

One of the features most consistently shown to be associated with obesity measures is temporal impulsivity^1,2^, a subdomain of general impulsivity^3,4^. It can be measured with a delay discounting (DD) paradigm, which tests whether participants prefer smaller, immediate rewards to larger, delayed rewards. Obese individuals tend to choose impulsively on this paradigm, showing more choices for immediate rewards than lean individuals^5,6^.

Value-based decision-making, and DD in particular, engages the dorsolateral prefrontal cortex (dlPFC), ventromedial prefrontal cortex (vmPFC), and striatum amongst others^7–10^. In healthy participants, choosing delayed rewards in DD has been shown to be related to activity in the dlPFC^9,10^. In addition, the subjective value of rewards in those tasks has been shown to be reflected within valuation regions^11^ of the vmPFC, the ventral striatum, and the posterior cingulate cortex^8,12–15^. The value signals in the vmPFC are then thought to be modulated by the dlPFC depending on the context^11,16^. This is supported by findings that effective connectivity between vmPFC and dlPFC predicts individual discount rates^17^. Relatedly, the dlPFC activation and connectivity is altered in obese participants while viewing problematic stimuli^11,18–20^ – food pictures, gustatory or olfactory cues – and in tasks requiring cognitive control^20–22^. This difference is associated with lapses in exerting control over eating behaviour in tempting situations^21,23,24^, and might thus be associated with increased DD behaviour.

Research shows that DD behaviour is malleable and depends largely on the framing of the two monetary options, paying attention to the delay vs. the reward, time frames in which the options are available, or even framing options with decimal numbers vs. without them^15,25^. It can also be influenced by stress, mood, affective stimuli^25^ or incidental cues^26–28^ – cues not associated with the decision-making process at hand. It has been shown that cues of positive valence generally primed participants towards choosing more immediate options^26,29^, while the opposite effect was shown for negative cues^28^. However, little is known about incidental priming in obesity. We are aware of only one study that addressed this issue^30^. Here, obese participants showed stronger priming than lean participants on the DD paradigm using visual stimuli of positive valence. This effect was mediated by sex, as obese men chose more immediate options, whereas obese women chose more delayed options. Negative cues were never tested in this context, and it also remains unclear whether positive cues would still have the same effect when both negative and positive cues are presented in the same paradigm.

In this study, we further explored the observation that obese participants are more susceptible to priming. We used a DD paradigm to investigate the influence of food stimuli on decision making in a general, food-independent context. The reason for this was that it is unknown from the current literature to which extent environmental food cues, which are thought to only exert an effect on dietary decisions, might generalise to other modalities, such as financial ones. Such an influence has been shown before for a variety of stimuli, ranging from brand logos to foods (e.g.^26,30^), and there is further evidence that the brain automatically extracts specific image features related to decision-making even when presented as an incidental, task-irrelevant background image (e.g.^31,32^). If, as suggested before^30^, obese individuals are indeed more susceptible to such primes and generalisation effects, this would point to an even greater vulnerability to undesirable decision biases, but potentially also shed light on general distorted cognitive processes in obese individuals. Using food-related incidental cues allowed us to investigate whether and how these stimuli affect general, food-independent decision making processes in obesity, which regularly occur in real-world environments. We therefore tested whether food cues can be used to alter disadvantageous decision making processes in obesity.

Using fMRI, we investigated a potential differential engagement of the dlPFC, striatum and vmPFC in lean and obese participants during DD decisions, and a potential modulation of decision-related brain activity when participants were exposed to incidental priming. We used visual (remote food prime) and gustatory (proximal food prime) cues of positive, negative and neutral valence. Based on a previous study, we expected that negative cues would elicit more priming towards delayed options, whereas positive cues would have an opposite effect^28^. Based on the assumption that remote cues signal *potential* food intake, i.e. availability of food, and proximal cues signal *acute* food intake, i.e. with direct consequences to the body, we expected stronger effects in the proximal condition. Further, we hypothesised the priming effect to be larger in obese participants^30^, and that this enhanced effect would be expressed in changes of brain activity in the dlPFC, vmPFC (increased activity for delayed choices) and striatal areas (increased activity for immediate choices^8,10,11^). This hypothesis was also based on previous literature showing changes in the dlPFC and vmPFC activity in similar paradigms utilising incidental cues in DD paradigms^26,28^. Previous reports show differences between obese and lean participants in the dlPFC activity in a number of tasks^20–22^, which are related to changes in cognitive control. It is therefore reasonable to assume that increased DD in obesity is also related to altered function of the cognitive control system, meaning that it can be hypothesised that differences in discounting behaviour between obese and lean people should be reflected in differences in the activation profile of the dlPFC. Moreover, since the priming effect was hypothesized to change the subjective values of delayed rewards, we expected that it would be associated with changes in activity of and connectivity between valuation regions.

## Materials and Methods

### Participants

56 healthy lean and obese participants aged 18–35 years took part in our experiment (29 men in total, subsample 1: 30 lean, mean BMI = 22.14 kg/m^2^, SD 1.81; subsample 2: 26 obese, mean BMI = 34.32 kg/m^2^, SD 3.37; group differences in BMI: t(54) = −16.499, p = 0.019; lean: mean age = 25.83 years, SD = 3.14, obese: mean age = 27.42, SD = 4.16, group differences in age: t(54) = −1.627, p = 0.110, see Supplementary Table S1, and supplementary section Participants for more details on demographics and recruitment procedures). BMI criteria for inclusion in the lean group: 18–25 kg/m^2^, obese group: BMI above 30 kg/m^2^. Participants met the following *a priori* inclusion criteria: no history of neurological/psychological diseases, no drug, cigarette or alcohol addiction, no hypertension or diabetes, no MRI-related contraindications. The full fMRI sample was reduced to 51 participants due to technical problems during the fMRI session (for exclusions see section Data analyses). Volunteers were compensated for taking part in the experiment with 7 Euro/hour for behavioural sessions and 8 Euro/hour for MRI sessions. The study was conducted according to the Declaration of Helsinki and approved by the Ethics Committee at the University of Leipzig. All participants gave their written informed consent prior to their participation in the study.

### Procedures overview

#### Behavioural task

We invited participants to the experiment twice: first, to a behavioural session, second, to the MRI session. The second session was conducted not later than 7 days after the first session. The behavioural session consisted of an introduction to the experiment, a set of questionnaires (see supplementary materials section Questionnaires for details on questionnaires used) and a computerised DD task. During the task, we offered participants two hypothetical monetary options, one smaller but immediately available reward (SIR) and one larger, delayed (available after a variable delay of 1, 2, 4, 6, 9 or 12 months) reward (LDR). The task was closely aligned to the procedures described in Simmank and colleagues’ study^30^. The two-step procedure included a ‘dynamic adjustment task’ and a ‘random choice task’. This session provided us with estimates of individual indifference points (IPs), which indicate the point of statistical indifference between immediate and delayed options. In other words, the IPs were defined as the ratio of SIR to LDR for a given delay where the subjective value (SV) of LDR was equal to the value of SIR. In the ‘dynamic adjustment task’, the IPs were calculated using a staircase procedure, where participants were offered different immediate and delayed rewards. This was done until two consistent IPs were obtained for each of the delays. Further, the ‘random choice task’, in which combinations of the previous task were administered again in a random order, was used to validate parameters obtained in the ‘dynamic adjustment task’. The obtained IPs were then used to define individualized *difficulty parameters* for the fMRI session. This parameter defines a ratio of SIR to LDR and was obtained by multiplying the IPs by one of twelve values (0.10, 0.15, 0.65, 0.75, 0.85, 0.95, 1.05, 1.15, 1.25, 1.35, 1.85, 1.90), thus returning twelve individual *difficulty parameters*, which were used to create the choice pairs. This was done in order to account for individual baseline differences in delay discounting and to ensure that the probability of choosing the LDR was similar for each participant in neutral conditions, making the combinations comparable between participants (the success of this approach was indicated by lack of group differences in probability of choosing the LDR in the neutral priming conditions). The *difficulty parameter* values indicate difficulty of intertemporal choice, with values close to 1 corresponding to more difficult choices (where SIR and LDR have similar subjective values), and values largely different from 1 corresponding to easier choices (SIR and LDR having different subjective values).

#### fMRI task

**Figure 1:**
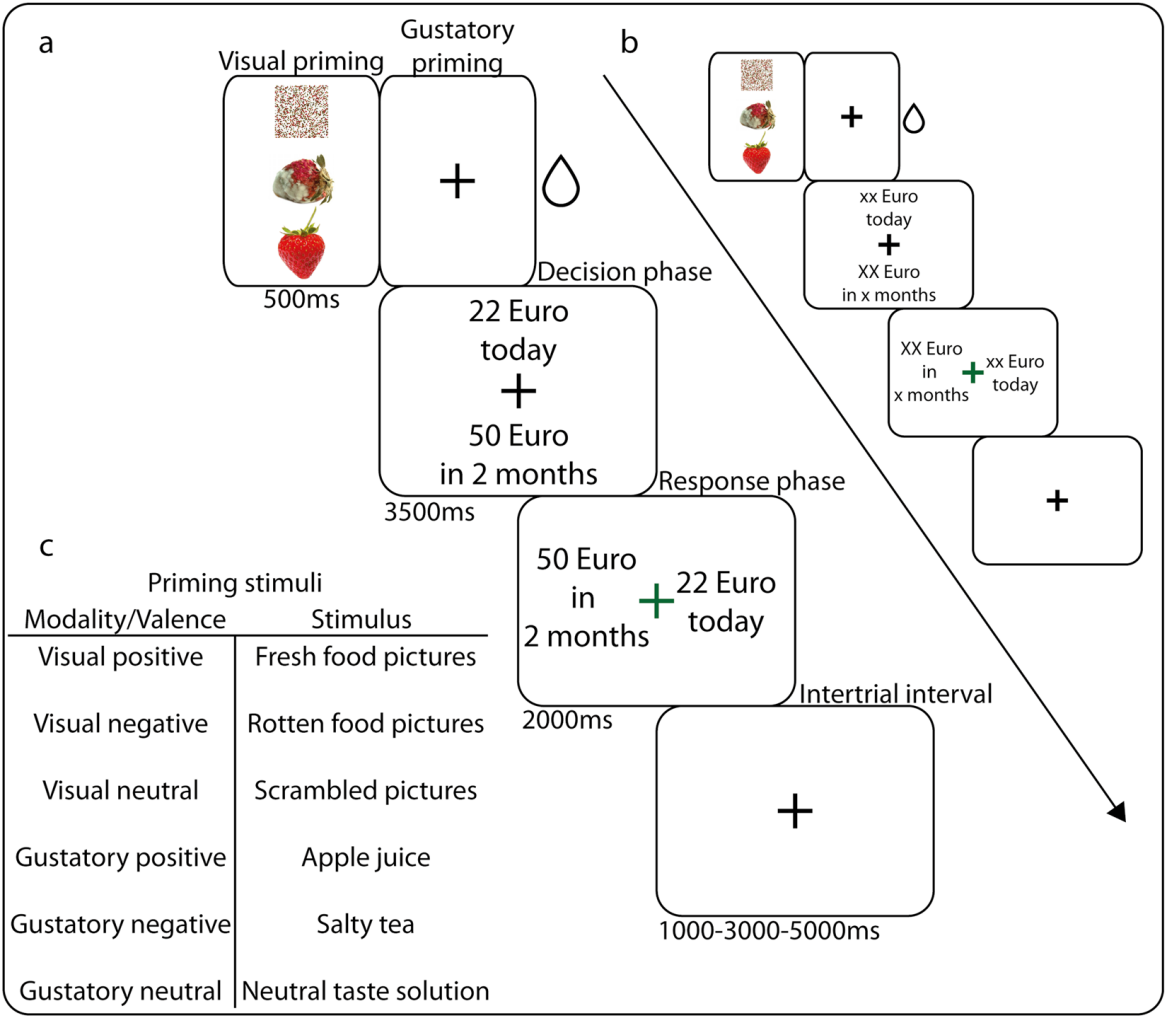
Overview on the experimental paradigm. (**a**) Delay Discounting (DD) and (**b**) Perception (P) trial outline. Each trial consisted of a priming period (500 ms), task screen (3500 ms), response screen (2000 ms) and a jittered intertrial interval (1000-3000-5000 ms, logarithmic distribution). During the priming period, a picture (visual conditions)^33^ or a fixation cross (gustatory conditions) was presented on the screen. In the gustatory conditions, taste liquids were delivered to participants simultaneously, for 500 ms. The task screen consisted of a fixation cross and the two hypothetical rewards presented above and beneath it (random placement). For the response screen, the fixation cross turned green and the rewards appeared on its left and right sides (random placement). Not presenting the choice options as mapped to left and right buttons during the initial presentation phase allowed preventing any early motor preparation processes from occurring, which would have confounded the neural signals reflecting decision making. Visual stimulation during the intertrial interval consisted of a fixation cross. (**c**) list of priming stimuli used in our experiment.

The fMRI part of the experiment consisted of ‘primed delay discounting’ trials and ‘perception only’ trials mixed and presented in a random order (Fig. 1). Gustatory trials were presented alternating with visual trials, hence one condition was never directly repeated. The total number of trials per experiment was 384. Participants indicated their choices by pressing buttons on single button boxes that were placed in each of their hands. This part of the MRI experiment lasted approximately 54 minutes and was divided into four blocks with breaks in between. After completing the experiment, participants evaluated the priming stimuli (described below) on a visual analogue scale from 0–100 (negative-positive). To make the hypothetical choices more realistic, we offered participants a 1/6 chance of winning one of the rewards they chose during the ‘primed delay discounting’ trials. The reward was chosen at random, and the monetary amount was either added to the participants’ reimbursement (immediate choice), or transferred to their bank account after a delay (2 months) corresponding to their choice.

Primed delay discounting trials: Before making each DD decision, we presented participants with one of six different food-related stimuli (DD conditions, Fig. 1). We used visual and gustatory stimuli of positive, negative and neutral valence, thus introducing 6 different DD conditions (gustatory positive – G_pos_, gustatory neutral – G_neu_, gustatory negative – G_neg_, visual positive – V_pos_, visual neutral, V_neu_, visual negative – V_neg_). Positive and negative food pictures were acquired from the FRIDa database^33^ (see Figs S1 for stimuli used). We chose positive and negative food pictures according to their ratings and content, creating six pairs of valence-matched images with the same content (e.g. positive bread – negative bread, Table S2). To create neutral visual prime stimuli, the valenced pictures were divided into a matrix of 53 × 53 100-pixel squares and subsequently randomly scrambled. This step was performed to preserve the colour composition of visual stimuli at the same time erasing their content. The gustatory stimuli were apple juice (positive), salty tea (negative) and neutral taste solution (neutral^34^, see supplementary materials section Administration of gustatory stimuli for details on stimuli administration). A visual trial was always followed by a gustatory trial, and *vice versa*. To reduce the duration of the experiment we used only one temporal delay for all trials (2 months) in combination with 12 pairs of SIR and LDR (corresponding to 12 levels of difficulty). Each of the pairs was repeated four times per condition in a random order (participants did not notice the repetition). Thus, the number of trials in the DD conditions was 288.

Perception only trials: To control for perception-related activity in priming-related brain activity, we introduced six analogous ‘perception only’ conditions with the same priming stimuli as in the DD conditions. Here, instead of the hypothetical rewards and a delay, participants were shown letters X or x and were asked to press a button corresponding to the side on which the capital letter X appeared (P conditions, Fig. 1). Each P condition was repeated 16 times, which amounted to 96 trials.

### Neuroimaging

The neuroimaging data were acquired using a 3 T Siemens PRISMA scanner with a 32-channel head coil. 1520 T2* images were collected using an EPI sequence (TE = 22 ms, FA = 90^o^, TR = 2110 ms, 40 slices, voxel size: 3 × 3 × 3 mm) over a time of 54 minutes. Each image was acquired in an ascending fashion. For 31 participants, whose anatomical images were not available in the Institute’s database, we acquired high-resolution MPRAGE images (TE = 2.98 ms, FA = 9^o^, TR = 2300 ms, TI = 900 ms, voxel size: 1 × 1 × 1 mm). There were 25 participants whose anatomical images were available through the Institute’s database. For those participants the time between anatomical image acquisition and the current experiment was: 3 years for 2 participants, 2 years for 8 participants, 1 year for 8 participants, and images were acquired the same year as the current experiment for 7 further participants. This sample of 25 participants included 18 lean and 7 obese individuals.

### Data analyses

#### Sample sizes

The full sample size for analysis of behavioural, baseline delay discounting and questionnaire data was n = 56. The final fMRI sample for analysis of perceptual-related, choice value-related and task-related choice-independent brain activity was n = 51. For behavioural and fMRI priming analyses and task-related choice-dependent fMRI analyses we had to exclude participants who chose either exclusively immediate or delayed rewards (*post hoc* exclusion criterion), and outliers concerning the cumulative priming effect for each of the four non-neutral priming conditions. The outliers were *a priori* defined as values lying more than 1.5 interquartile range above/below Tukey’s hinges (H1 and H2). This resulted in a final sample size of n = 36 participants (19 lean, 17 obese).

#### Behavioural data

Behavioural data were analysed using SPSS 22 (IBM, Armonk, New York, United States, statistical analysis of behavioural data) and MATLAB 2012b (The MathWorks, Inc., Natick, Massachusetts, United States, modelling of delay discounting data using quasi-hyperbolic model). To investigate between-group differences in the ratings of the priming stimuli, we used an ANOVA with weight status as a between subject variable. To test for rating differences between stimuli of different modalities and valences, we used a repeated measures ANOVA with modality and valence as within subject factors. Baseline and primed delay discounting data were plotted using ggplot2 toolbox for R in RStudio^35–37^.

#### Delay discounting data modelling

Following Simmank and colleagues^30^, who tested different discounting models for a similar task, we assumed that the delay discounting behaviour is represented by a quasi-hyperbolic model defined by:

$${\rm{\alpha }}={{\rm{\beta }}{\rm{\delta }}}^{{\rm{T}}},$$

where alpha is the subjective value of a delayed reward, beta is a delay-independent bias towards immediate rewards, delta is a delay-dependent discount factor, and tau is the delay^38^. Simmank and colleagues have used a similar sample of lean and obese participants and, to our knowledge, are the only ones to have specifically compared the fit of hyperbolical and quasi-hyperbolical models to the delay discounting data. Therefore, we decided to follow their recommendations and use the quasi-hyperbolic model as well. For the behavioural part of the experiment, choices in each individual trial were entered into the model and beta and delta parameters were calculated (for details see ‘Estimation of Discount Function’ section in Simmank *et al*.^30^). We investigated group differences in the DD parameters using a general linear model. We included weight status and sex as fixed factors, and the obtained professional degree as a covariate in the model (due to significant between-group differences). We decided to use sex as a factor because an earlier study showed sex-dependent differences in DD between obese and lean participants^6^.

#### Primed delay discounting data modelling

In order to obtain a trial-by-trial measure of the priming effect, we calculated the probability of choosing the delayed reward during each trial by fitting a generalized linear model with a logit link function to the data. Here the 12 *difficulty parameter* values represented a predictor variable, and the dependent variable was denoted by the actual probability of choosing the LDR extracted from the data. We then extracted individual probabilities of choosing LDR for each *difficulty parameter* from the model. To obtain a comparable measure of the priming effect, we subtracted the probabilities for neutral conditions from the positive and negative conditions within respective modalities, thus obtaining four separate measures, one for each non-neutral priming condition.

To investigate whether probabilities of choosing the LDR were different for each *difficulty parameter*, we entered the priming effect values for all *difficulty parameters* into a repeated-measures general linear model as a within subject, dependent factor. This was done separately for each of the four non-neutral priming conditions.

Further, we aimed to establish whether the priming effect for different conditions, independent of *difficulty parameter*, was different from 0 and different between groups. For this, we calculated a cumulative priming effect for each condition by adding all the individual probabilities of choosing the LDR. We then used a one-sample t-test to investigate whether the cumulative priming effect in each condition was different from zero. This would indicate a significant change of probability of choosing the LDR from a neutral condition to the corresponding positive or negative priming conditions. We used a two-sample t-test to investigate whether there were any between-group differences between priming conditions. Previous studies showed quadratic associations of responsivity to reward and the physiology of the reward system with BMI^20,39–42^. We therefore tested whether the cumulative priming effect was related to BMI^2^.

Following our findings on the behavioural level and to mimic our neuroimaging analysis (GLM 6), we decided to investigate whether BMI and BMI^2^ influence *difficulty parameter*-dependent priming in the G_neg_ condition. To this end we entered priming effect values for all *difficulty parameters* into a repeated-measures general linear model as a within-subject, dependent factor, and BMI and BMI^2^ as covariates^43^.

#### Neuroimaging data analysis

We used FSL 5.0.8 (The University of Oxford, Oxford, United Kingdom), SPM 12 (Wellcome Department of Cognitive Neurology, London, United Kingdom) and MATLAB R2012b to pre-process and statistically analyse the functional imaging data. Brain figures were plotted using Nilearn. Anatomical structures corresponding to peak voxels were identified using the xjView toolbox (http://www.alivelearn.net/xjview). To enable further pre-processing steps, structural images were skull-stripped using FSL’s brain extraction tool^44^ and SPM12 segmentation tool. Functional data were motion corrected using McFLIRT^45^, slice-timed, and smoothed with a 6 mm FWHM Gaussian Kernel, and normalised to MNI space using FSL. To remove motion and physiological noise-related artefacts, we used an automatized independent component analysis approach (ICA-AROMA)^46^. Prior to statistical analysis on an individual level, the data were high-pass filtered with a filter of 128 s. Each step of the pre-processing pipeline was quality checked (visual inspection of the data).

#### Analysis of BOLD response

A two-level group random effects analysis was performed using SPM12. Single subject regressors were entered into a univariate general linear model and convolved with a double-gamma hemodynamic function. Individual contrast files were then entered into a second level analysis (GLM1: flexible factorial model, remaining GLMs: one- or two-sample t-tests). In this step, BMI, age and sex were entered as covariates-of-no-interest, in order to control for variance in those variables. Additionally, for analysis of priming effects in the G_neg_ condition, we added BMI^2^ as a covariate of interest (see results section Primed delay discounting). Unless stated otherwise, presented results were thresholded at a whole-brain voxel level with an *a priori* threshold of p < 0.005 and corrected for multiple comparisons on a cluster level with p < 0.05 (family-wise error, FWE). The FWE p-value was Bonferroni corrected for the number of GLMs used in the study, resulting in an effective p-value threshold of 0.007. In addition to using these conventional thresholds, we also tested whether our results survive a more stringent whole-brain voxel level threshold of p < 0.001 (using an FWE correction at p < 0.007). Wherever applicable, the information that results were still significant when using this threshold is stated in the text and in corresponding Tables.

First, we modelled brain activity related to perception and priming-independent choice (see supplementary materials GLM 1, GLM 2 and GLM 3 sections for details of those analyses) in order to replicate findings already reported in the literature. GLM 4 and GLM 5 were the models of main interest, as they investigated how brain activity is related to priming effects that we observed on the behavioural level. GLM 6 and GLM 7 were parts of a *post-hoc* analysis to elucidate mechanisms behind the main effects.

Including all regressors of interest in one GLM would have required including more than 200 regressors. Hence, some of the analyses needed to be separated into different models. Moreover, this approach allowed us to maximise statistical power for our analyses, as some analyses (without finer distinctions between conditions) could be conducted within the full fMRI sample (51 participants, GLM 1 and GLM 2), instead of with the priming sample (36 participants, GLMs 3–7).

GLM 4: Priming related changes in brain activity: We investigated whether behavioural priming effects were reflected in changes in brain activity. Here, we entered 14 regressors into the first level analysis: 12 reflecting 12 different priming conditions (see task description above for details), irrespective of the delayed or immediate choice, and 2 regressors reflecting the response phase and the priming phase (independent of the condition). This analysis was used to investigate how brain activity in the G_neg_ condition differed from the other conditions, since we only found significant behavioural effects in this condition. This was based on *a priori* assumptions that we would only investigate brain activity differences in conditions for which behavioural effects were significant.

GLM 5: Trial-by-trial priming effect modulation of brain activity: To investigate whether brain activity was modulated by the trial-by-trial priming-effect (the probability of choosing the LDR in a priming condition minus the probability of choosing the LDR in a respective neutral condition) in the G_neg_ condition, we entered the priming effect as a parametric modulator of a regressor containing all DD trials within this condition (unlike GLM 4). As described above, this regressor was obtained by modelling *difficulty parameter-*dependent probabilities of choosing delayed rewards for each individual trial. Additionally, we entered 13 other regressors – 11 for each of the remaining conditions (in which no significant priming effect was found), and two other regressors representing the priming phase and the response phase (independent of the condition). The contrast of interest was the parametric regressor.

GLM 6: Psychophysiological interaction (PPI) analysis: In order to better understand the mechanisms underlying the observed priming effect, we performed a PPI analysis. This analysis describes how contributions of different brain areas to each other change within a psychological context^47^. Since those interactions are assumed to occur on a neural level, not on the hemodynamic level, PPI involves steps such as deconvolution of the BOLD signal, calculating interaction term with the psychological variable, and reconvolution of this interaction with the hemodynamic response function. Here, we tested whether the dlPFC (hypothesised and identified in GLM 4, see Results) would change its connectivity with other brain regions depending on the trial-by-trial probability of choosing the LDR that we entered as a parametric modulation into the model. For this analysis, we used peak voxel coordinates from the GLM 4 contrast of the gustatory negative conditions versus other conditions (as this condition revealed the strongest results, see below), and defined a 6 mm radius sphere around it as a volume of interest. We then created a separate GLM, which consisted of 16 regressors: time course of the VOI (physiological factor), values of parametric modulation (trial-wise probability of choosing the delayed reward; psychological factor), the PPI term, and 13 remaining regressors representing 11 priming conditions (without G_neg_) and response and priming phases of the experiment (independent of condition). This was done according to recommendations by O’Reilly and colleagues^48^. In comparison to the GLM 5, GLM 6 includes three additional regressors specific for the PPI analysis, and no regressor representing the G_neg_ condition_._ The second level contrasts of interest included the PPI interaction regressor, and the BMI regressor. The first contrast represented functional connectivity differences related to the priming effect, while the second represented correlation of BMI with connectivity differences dependent on the priming effect.

GLM 7: PPI analysis: group differences in G_neg_ condition: In this model we aimed to investigate general group differences in connectivity between gustatory negative and gustatory neutral conditions independent of choice difficulty (based on behavioural group differences). To do that, we used the same seed regions as in the GLM 6 analysis and created three PPI regressors: time course of the VOI, main effect regressor (G_neg_ > G_neu_, psychological factor), and the PPI term. Similarly to GLM 6, we added 10 regressors to the model representing the remaining priming conditions and 2 representing response and priming phases of the experiment. Here, contrast of interest was the group difference between PPI interaction regressor, representing connectivity differences between the G_neg_ and G_neu_ conditions.

## Results

### Income and education

Lean participants had higher academic education than obese participants. Moreover, obese participants tended to earn more than lean participants. Differences in income might influence delay discounting behaviour, however, in a way where participants earning more money should have lower delay discounting^49^. In our sample, however, obese participants earned more and were steeper discounters.

### Priming stimuli ratings and questionnaire correlations

In short, we found group differences in ratings of stimuli only in the visual negative conditions (less negative ratings for obese individuals; Table S4). Further, we found rating differences for valence and modality for both groups pooled (Table S5) – negative stimuli were rated as most negative and positive as most positive, and visual stimuli were generally rated lower than gustatory. Regarding questionnaire measures, we found significant group differences in the Cognitive Restraint subscale of the Three Factor Eating Questionnaire, and no significant correlations between delay discounting parameters and questionnaire measures (Table S6).

### Baseline delay discounting differences

Here we analysed group differences in baseline delay discounting. The results show group differences in the delay-independent parameter (F(1, 51) = 4.140, p = 0.047), and a group by sex interaction for the delay-dependent parameter (F(1, 51) = 5.736, p = 0.020, see supplementary materials section Baseline delay discounting and group differences, Fig. S2, and Table S7 for more details on the results).

### Primed delay discounting

**Figure 2:**
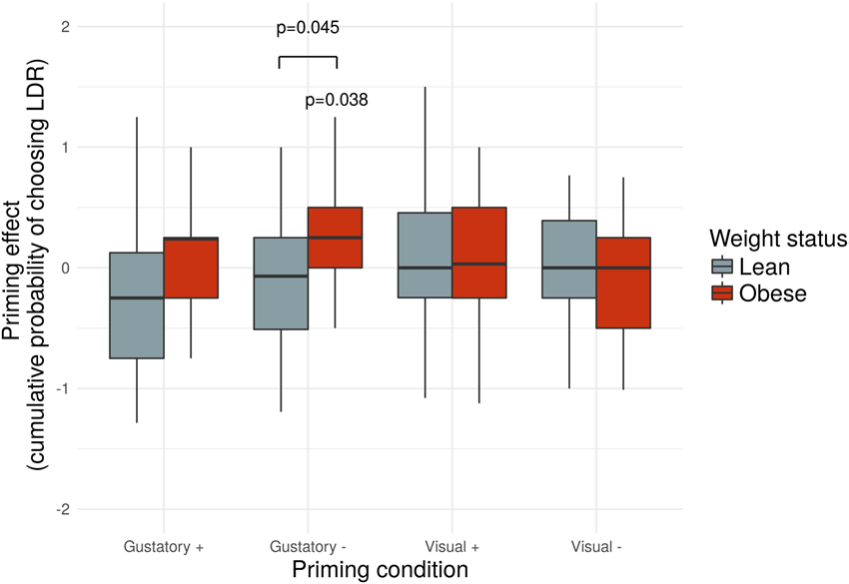
Condition dependent priming effects plotted separately for lean and obese group (N = 36). We found that the cumulative priming effect in the gustatory negative condition was significantly different from zero in the obese group (p = 0.038), which means that here participants chose more delayed options than in the gustatory neutral condition; additionally, we observed group differences in the gustatory negative condition (p = 0.045), indicating that obese individuals were primed more towards delayed choices than lean individuals. The bold horizontal line represents sample**’**s median, horizontal lines below and above represent 1^st^ and 3^rd^ quartiles, and the whiskers represent minimum and maximum data points.

We showed that the priming effect depends on choice difficulty in the G_neg_ condition only (main effect of group within the G_neg_ condition: F(11, 19) = 2.222, p = 0.048; Table 1). This result points to the fact that choice difficulty (difference between SIR and LDR) is a factor influencing priming effects within the G_neg_ condition. Moreover, the cumulative effect of priming was different from zero only for the obese group in the G_neg_ condition (t(16) = 2.263, p = 0.038; Table 2, Fig. 2). This indicates that the obese group was primed towards more delayed choices during this condition relative to G_neu_ condition. Further, there was a significant difference in the priming effect in the G_neg_ condition between the lean and obese groups (t(34) = 2.080, p = 0.045; Table 2). Following up on this, we tested for a direct relationship between BMI and the cumulative priming effect in the G_neg_ condition. We additionally tested for a quadratic relationship between the priming effect and BMI. Indeed, there was a quadratic relationship between BMI and the cumulative priming effect (R^2^ = 0.208, p = 0.021, see supplementary Figs S6 for details on the distribution and correlation). Due to this, we added BMI^2^ as a covariate in the fMRI analyses involving priming effects in the G_neg_ condition (GLM4–7). Additionally, to mimic our neuroimaging analysis, we performed a *post hoc* repeated measures ANOVA with *difficulty parameter*-dependent priming effect values within the gustatory negative conditions as dependent variables and BMI and BMI^2^ as covariates. This analysis showed that BMI and BMI^2^ are significant predictors of choice difficulty-dependent priming in the full sample, including both lean and obese participants, in the G_neg_ condition (BMI: F(11, 23) = 3.035, p = 0.012, BMI^2^: F(11, 23) = 4.254, p = 0.002).

**Table 1:** Multivariate statistics showing differences in priming effect depending on the *difficulty parameter* (N = 36).

Condition	F value	p value	Partial η^2^
Gustatory positive	F (11, 19) = 1.648	0.146	0.420
Gustatory negative	**F (11**, **19)** **=** **2**.**222**	**0**.**048**	**0**.**494**
Visual positive	F (11, 19) = 1.065	0.425	0.319
Visual negative	F (11, 19) = 1.188	0.344	0.343
Gustatory negative with BMI and BMI^2^ as covariates	**BMI: F (11**, **23)** = **3**.**035 BMI**^**2**^**: F (11**, **23)** = **4**.**254**	**BMI: p = 0**.**012 BMI**^**2**^**: p = 0**.**002**	**BMI: 0**.**592 BMI**^**2**^**: 0**.**670**

**Table 2:** Cumulative effect of priming (independent of *difficulty parameter*, N = 36).

Condition	Lean		t(18) value	p value	Effect size |d|	Obese		t (16) value	p value	Effect size |d|	Lean vs. Obese		
	Mean	Standard deviation				Mean	Standard deviation				t(34) value	P value	Effect size |d|
Gustatory positive	−0.21	0.76	−1.186	0.251	0.276	0.08	0.47	0.730	0.476	0.170	−1.357	0.184	0.453
Gustatory negative	−0.13	0.63	−0.921	0.369	0.206	0.25	0.46	**2**.**263**	**0**.**038**	**0**.**544**	**−2**.**080**	**0**.**045**	**0**.**683**
Visual positive	0.09	0.62	0.635	0.534	0.145	0.01	0.66	0.119	0.907	0.015	0.333	0.741	0.125
Visual negative	−0.03	0.61	−0.205	0.840	0.049	−0.09	0.52	−0.205	0.493	0.173	0.314	0.755	0.105

One-sample t-test against zero and two sample t-test for group comparison. The cumulative effect of priming was different from zero within the gustatory negative condition for the obese group. Obese participants showed more delayed choices than lean participants in the G_neg_ condition.

### Neuroimaging results

#### Perceptual, task-related and choice value-related brain activity

Results from GLM1 – GLM3 concerning perception-related brain activity, choice-value related brain activity and task-related brain activity can be found in supplementary materials in corresponding sections, Figs S3–S5, and Tables S8–S10. In short, we found gustatory-related brain activity in the bilateral Rolandic operculum, bilateral insula and medial frontal gyrus, and visual-related brain activity in the bilateral fusiform gyri. DD trials, as opposed to P trials elicited activity in the frontal, occipital and parietal regions, while immediate choices were related to increased activity in the middle frontal gyrus, medial frontal gyrus, cerebellum and cingulate gyrus. Choice value during the task was tracked by the medial frontal gyrus.

#### Priming-related brain changes

**Figure 3:**
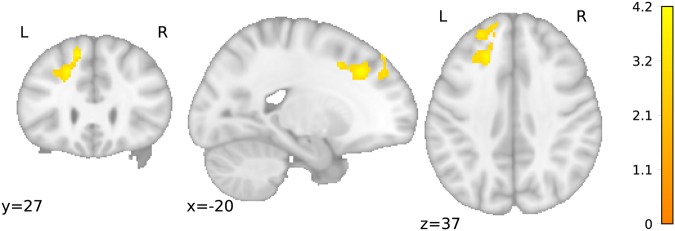
Brain region where brain activity during gustatory negative priming is higher for lean than for obese participants. L – left, R – right. T-values are plotted on a standard brain (N = 36, cluster defining threshold 0.005, p_FWE_ = 0.007).

This analysis was aimed at investigating how brain activity differed during priming. Since the behavioural differences were found only for the G_neg_ condition and in the obese group, we only investigated group differences in this condition. Using a two sample t-test we found that brain activity (DD trials > P trials) in the left superior frontal gyrus was lower for obese than for lean participants in the G_neg_ condition in relation to all other conditions (Table 3, Fig. 3). Specifically, we computed single subject contrasts by assigning a positive weight to the G_neg_ condition and negative weights to other conditions. Here, our findings imply a role for this region in mediating the priming effect.

**Table 3:** Brain region associated with priming effects in the gustatory negative condition (N = 36).

Contrast	Region of the peak voxel	Cluster size [voxels]	Coordinates (MNI)			Peak z score	Peak t score
Obese < lean for G_neg_ condition versus all other conditions	Superior frontal gyrus L	738	−20	28	38	3.70	4.21

R – right, L – left.

#### Trial-by-trial priming effect modulation of brain activity

In this analysis we investigated whether brain activity in the gustatory negative condition was parametrically modulated by the priming effect. For this analysis we did not find any statistically significant results.

#### Priming-related PPI connectivity changes

**Figure 4:**
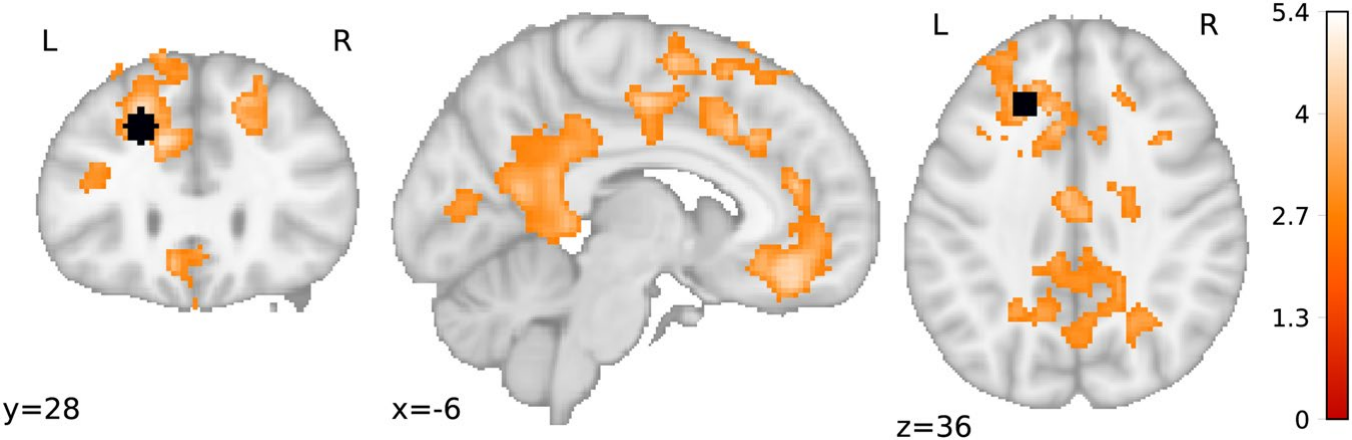
Brain regions where PPI connectivity with the dlPFC modulated by the trial-wise priming effect correlated negatively with BMI. L – left, R – right. Black shape depicts the seed region. T-values are plotted on a standard brain (N = 36, cluster defining threshold 0.005, p_FWE_ = 0.007).

While the general analysis for trial-by-trial priming effects was not significant, another possibility was that not the average activation but functional connectivity of priming related regions was modulated by the priming effect. We further hypothesised that such connectivity changes might additionally be modulated by BMI. This logic is in line with our behavioural results, where priming effect in the G_neg_ condition is related to different BMI values (see section 3.4). Our PPI analysis confirmed this hypothesis and showed a negative correlation of BMI with connectivity modulated by the trial-wise priming effect between the left superior frontal gyrus (as the seed region) and regions of the left middle and superior frontal gyri, precuneus and medial frontal gyrus (Table 4, Fig. 4, Supplementary Figs S7–S12). Clusters surviving a more stringent voxel-wise threshold of 0.001 are denoted in Table 4. These results imply that for the G_neg_ condition higher BMI and higher priming effects were related to lower connectivity between left superior frontal gyrus and these regions. They are also in line with our behavioural results showing that BMI is a predictor of *difficulty parameter-*dependent priming effect.

**Table 4:** Brain regions whose connectivity with the left superior frontal gyrus correlates negatively with BMI and is modulated by the trial-wise priming effect within the gustatory negative condition (N = 36).

Contrast	Region of the peak voxel	Cluster size [voxels]	Coordinates (MNI)			Peak z score	Peak t score
BMI correlation with connectivity modulated by the trial-wise priming effect	Middle frontal gyrus L*	4023	−24	22	44	4.50	5.40
	Cerebellum R	1315	42	−76	−32	4.14	4.85
	Occipital lobe L*	5580	−36	−66	−2	4.08	4.74
	Medial frontal gyrus L*	939	−6	40	−14	3.97	4.57
	Postcentral gyrus L	688	−54	−22	56	3.89	4.46

R – right, L – left; * denotes clusters surviving 0.001 voxel-wise threshold and further FWE correction (0.007).

We observed no general group differences in PPI analysis of G_neg_ and G_neu_ conditions (GLM 7).

Figures depicting contrast estimates and 95% confidence intervals for peak voxels in each fMRI analysis can be found in supplementary Fig. S13.

## Discussion

In this study we investigated whether 1) obese participants showed higher delay discounting than lean participants, and 2) whether they were more susceptible to incidental priming on the delay discounting paradigm than lean individuals. In a second step, we investigated the neural correlates of any significant behavioural effects. We hypothesised that participants with obesity would show higher DD and higher susceptibility to priming. We further hypothesised that this higher susceptibility to priming would be reflected in differences in valuation (vmPFC), cognitive control (dlPFC), and reward related (striatum) brain regions. We showed higher baseline delay discounting for obese compared to lean individuals independent of delay. Moreover, group differences in delay-dependent discounting differed by sex. We also demonstrated a higher susceptibility to incidental priming on the delay-discounting task in obese participants (albeit only for one group of negative stimuli). Thus, our findings are in line with the most important findings regarding intertemporal decision-making in obese individuals^2,6,30,50^. In addition, the negative gustatory priming condition (contrasted against all other conditions) was associated with modulation of brain activity in the left dorsolateral prefrontal cortex (dlPFC), where we observed a lower activity for lean compared to obese participants. The priming effect was also indirectly related to dlPFC’s connectivity to the medial prefrontal cortex (mPFC).

Here, we replicated previous results showing higher delay discounting in obese compared to lean participants (for a meta-analysis and review see:^1,2^). We showed this for males and females by using a delay-independent discounting parameter. For a delay-dependent parameter, resembling the widely used *k* parameter of the hyperbolic discounting model, we found a significant interaction between weight status and sex. This differential effect of sex is in line with some previous studies showing changed DD only for females^5,6^, and in contrast to others^1,4,30,51–53^. Here, we used a quasi-hyperbolic delay discounting model which is different to most of the previous studies. It is conceivable that differences in parameter estimations between these models explain why others mostly did not find sex differences in their studies. Differences between individuals might also contribute to these finer differences in effects. There is no homogenous population of “obese individuals”, and hence fluctuations in variables not measured here, such as impulsivity, genotype or obesity duration, might have contributed to discrepancies in results between studies. However, given the diversity of findings so far, a systematic investigation focused on sex with large sample sizes would constitute a valuable future research program.

Taken together, our behavioural results provide evidence that obese participants were more impulsive in their financial decisions than their lean counterparts, but when primed by a negative food-related gustatory stimulus, this effect reversed. Simmank and colleagues^30^ interpreted their findings as evidence that obese individuals would be, more than others, biased in their general decision-making by different types of tempting, positive stimuli. As the decisions were not related to food, and the priming stimuli not related to the financial decisions, this could indeed point toward a general impulse control deficit in decision systems. Our results add to this view as we again show an effect of incidental stimuli on decisions in obese individuals, but this time reversed: While negatively valenced gustatory stimuli could be expected to delay the desire to consume food, these stimuli again had a transfer effect to delaying the desire to receive monetary rewards. Hence, our findings complement previous reports by showing that effects of incidental cues on decision-making might generalise, even for aversive stimuli. It is interesting to note that the positive gustatory priming, even though not significant, elicited a similar group difference pattern to the gustatory negative priming. However, this difference was mainly driven by the lean group, which showed a (non-significant) effect in the opposite direction towards more impatient choices, as expected from previous literature showing similar priming effects for a variety of positive cues^26,28,29^. However, since these findings did not reach significance in our study, this remains an observation that requires further investigation.

Incidental cues have previously been shown to effectively influence decision making processes in the context of delay discounting^26–28^. Our results are in line with the general findings by Simmank *et al*.^30^. Unexpectedly, however, in our experiment this phenomenon occurred in the gustatory negative condition only, in which obese participants showed stronger priming effects towards delayed choices. The direction of the effect is in line with the results of Luo *et al*.^28^. Simmank and colleagues^30^, on the other hand, showed a more diverse profile of obesity-sex interactions for a variety of visual stimuli. However, there were several important differences between these studies. First, Simmank and colleagues^30^ did not use gustatory stimuli, hence our results are not in conflict with their findings. Second, food-related cues were the only theme of all visual and gustatory stimuli in the present study while in the previous study a variety of positive visual stimuli, including social images, status symbols, and only a subset of food-related images, was used. This might have created strong framing and expectation effects. Third, in order to include all necessary control conditions and to maximise the number of delay discounting trials, we used only one temporal delay of two months, while Simmank and colleagues^30^ used several delays. This might have enabled participants to form expectations in our study and, in consequence, form rather stable indifference points. The resulting rather consistent choice patterns work against any potential priming effects. Supporting this conclusion, it should be noted that several participants, who had to be excluded from the analyses, exclusively chose delayed or immediate options in at least one condition, even though we initially tailored choice options to individual indifference points. Finally, a possible explanation for observing priming effects only in the gustatory negative condition is that our design was not optimised to detect priming effects and that only the strongest primes could impact on behaviour. Therefore, it is important to note that even under these suboptimal circumstances, obese participants were primed by gustatory negative cues. Although subjective valence assessments were not statistically different, these cues had nominally the most negative values for obese participants, which might offer an explanation for why this particular condition elicited effects on delay discounting behaviour. Another difference to previous work^30^ could be that our positive stimuli were not perceived as positive as intended. We did not find brain regions responding more strongly to positive than to negative stimuli, and the positive stimuli were also rated as being closer to neutral than the negative stimuli. The absence of effects for the positive stimuli should therefore be interpreted with care.

We further investigated how the behavioural priming effect in the gustatory negative condition was reflected in brain activity. Firstly, in the parametric modulation analysis of the G_neg_ condition by priming effect we found no significant results. The effects of G_neg_ condition on a trial-by-trial basis were very small, and our analysis might not have been sensitive enough to detect changes in brain activity related to these behavioural effects. It is also conceivable that trial-by-trial effect on the neural level was only reflected in connectivity changes, not in changes of brain activity. Further, generally during priming with negative gustatory stimuli, activity in the left dlPFC was significantly lower for obese than for lean participants. A very similar region of the dlPFC has previously been found to be related to perceptual decision-making^54–56^, suggesting a key role in integrating information relevant to make a decision, or in providing the cognitive resources required for making difficult decisions. These findings are in accord with our assumption that dlPFC is an important region for processing decision-relevant information for DD, and potentially for integrating the information contributed by the primes, and/or for modulating cognitive control in our task. Further, activity in the lateral prefrontal cortex (lPFC) has been shown to be related to more difficult choices, but also to choosing delayed rewards and exerting context-dependent control over behaviour^11,16,57–60^. However, opposing activation patterns^61–64^ or no activation patterns^7,8^ have also been reported. A theory posed by Weber *et al*. offers a possible explanation for these discrepancies^65^. They suggested that differences in reward discounting might be related to the query theory, which posits that individual preferences, also in value-based decision making, are determined by sequentially answering a number of internal queries regarding choice and order thereof^66–68^. Put simply, factors such as the order of reward evaluation, shift of attention to the reward’s magnitude or delay, and internal goals (answers to internal queries) influence delay discounting rates^25,65^. Applied to our results, this would mean that obese and lean participants might have differed concerning their initial internal goals, and therefore made different choices. It follows that neural mechanisms of maintaining these goals could then produce different response patterns. Different implicit goals of participants might explain differential engagement of the dlPFC in reward-related decision making. Therefore, the dlPFC might not only act as an inhibitory brain structure, constantly promoting less impulsive decision-making, but it could also promote more impulsive decision-making, provided that this is congruent with internal goals. In our study, obese compared to lean participants generally gravitated towards more immediate rewards, which could reflect intrinsic differences in internal goal structures. In consequence, priming towards delayed options in obese individuals was related to the observed *decrease* in dlPFC activity.

The results of our connectivity analysis point to a mechanism for how this goal-dependent activity of the dlPFC could influence behaviour. In our behavioural analysis we showed that BMI influenced the choice difficulty-dependent priming effect. We showed a similar effect in our PPI connectivity analysis. This indicated that during the gustatory negative condition connectivity of the dlPFC with the vmPFC, posterior cingulate cortex and parietal cortex was modulated by the trial-by-trial choice difficulty-dependent priming effect which, in turn, correlated negatively with BMI. These regions are discussed to be part of the default mode network (DMN)^69^. The DMN has previously been shown to be engaged in delay discounting. More specifically, in lean samples, activity within these brain structures has been shown to increase with choices of delayed rewards^8,10,70,71^. The vmPFC has also been shown to track trial-by-trial subjective value of chosen rewards (e.g.)^8^, an effect corroborated by our results. Thus, a modulation of the connectivity between the aforementioned brain regions might be necessary to alter decision-making processes. Indeed, two studies investigating neural correlates of primed intertemporal choices showed that the mPFC was directly related to the effects of priming with incidental cues^26,28^. Hare and colleagues further showed that dlPFC increased its connectivity to the vmPFC at the time of intertemporal choice, especially in trials in which participants chose LDRs^17^. Surprisingly, our findings suggest an opposite pattern for participants with higher BMI. This, however, might again be expected if one assumes that dlPFC activity depends on current individual goals. The increased connectivity in obese participants suggests that, by default, the dlPFC inhibits brain regions related to delayed choices and thus promotes immediate choices in obese individuals. During priming with negative gustatory cues, the brain regions promoting delayed choices may be disinhibited by means of inhibiting the dlPFC. However, it is important to note that the present PPI analysis does not include any information about the directionality of the connectivity effects between any of those brain regions. Therefore, this interpretation is speculative and warrants replication using different studies, methodology and analyses (e.g., using non-invasive brain stimulation approach).

Some potential limitations of the present study should be mentioned. Firstly, the proportion of excluded participants, in particular for some of the neuroimaging analyses, was relatively large. Some of these exclusions were necessary as we aimed to establish individual indifference points in the DD task, and for this choosing different options (as opposed to settling for one option only early in the experiment, as some participants did) was necessary. As in many other decision tasks, this task feature always comes with the risk that participants do not behave as expected. “Correcting” their behaviour by using additional instructions would bias the results and render them non-interpretable and had to be avoided by all means. Hence, while we recommend using an online adjustment of indifference points using a staircase function as a potential strategy for future studies, for our study excluding participants was the only option. We note that as a consequence, our sample size was lower than initially hoped, which is a limitation of this study and has to be taken into account when interpreting the results. Second, we did not explicitly test reliability of the DD parameters here (which can be a problem with DD tasks)^25^. However, previous work using a highly similar task in a comparable sample found very high re-test reliability for much longer time periods between sessions than here^30^, suggesting that this was not an issue for our study. Further, behavioural results in the priming section of our study were obtained by performing four independent tests. Correcting p-values for multiple testing (Bonferroni correction) results in nonsignificant findings. However, our overall sample size used in the priming analysis is sufficient, as we conclude from previous reports^26,28^, thus suggesting that the results are not just due to type I error. Generally, the behavioural effects we aimed to investigate in our study are quite small. Given the presence of the aforementioned limitations and the fact that we used a very ambitious design, the fact that we were still able to find a behavioural effect in one of the conditions (before correcting for multiple comparisons) is encouraging. Finding a neural correlate of this effect (with correction for multiple comparisons – which in the case of neural data is conducted for a multitude of tests and therefore extremely strict) provides a second step of validation for this finding.

Our neuroimaging results have been thresholded on a voxel-level of 0.005, and corrected for multiple comparisons on the cluster-level. Further, most of the results have survived a more stringent voxel-wise threshold of 0.001. Clusters found in remaining analyses, related to priming, but also to reward-valuation, are highly congruent with current literature. The vmPFC, which we found to correlate with trial-by-trail choice value, has been widely implicated in reflecting values of potential rewards^8^. Further, we hypothesised that the dlPFC and its connectivity to other brain areas would be related to priming. Thus, while we are aware of studies showing that the voxel-wise threshold of 0.005 might result in a higher rate of false positives^72^, we believe that the presented results are not merely due to type I error. It should be noted that there is also a problem with the probability of false negative results that is notoriously difficult to address in fMRI studies that rely on very specific groups of participants with natural sample size limitations, due to availability of participants (such as obese but otherwise healthy people)^73^. One suggested solution to this issue is to apply more liberal thresholding techniques, but also to incorporate strong a priori hypotheses concerning the location of relevant regions^74^ to guarantee sound and rigorous statistical testing. Altogether, we believe that our results show true effects, both on the behavioural and neuroimaging levels. We note, however, that independent replications would be desirable to confirm and expand on our results.

Our results have broader implications for impulse control in obesity as they show that decision making processes, beyond dietary decisions, are more easily influenced by food-related cues in obese individuals as compared to individuals without obesity. Importantly, observing such susceptibility to environmental cues on economic choices suggests that food cues may influence general decision making processes in obesity. Economic decisions are a substantial part of everyday life, ranging from smaller purchase decisions to decisions with potentially long-term consequences, e.g., whether to overspend and to accumulate debt, which is an increasing problem in our society. Priming susceptibility is especially important in light of today’s obesogenic environment, where food cues are ubiquitous, and might strongly influence all kinds of decisions in susceptible individuals. For example, food advertisements have been shown to trigger motivation to eat in obese participants^75^. We contribute to already existing literature on priming and delay discounting, which suggests that positive primes change behaviours towards less beneficial routines (i.e. less rational choices), while negative primes are said to have an opposite effect^26–30^. While it might be premature to draw strong conclusions from the limited number of studies showing enhanced priming effects in obesity about which primes have the strongest effects, our study nevertheless confirms the view that there is a general enhanced susceptibility to environmental cues in obese participants. This finding has implications for systematically targeting obese individuals (or potentially even individuals who might be at risk) with specifically designed cues. For example, a previous study using health warnings showed that negatively framed graphic warnings promoted higher self-control in dietary decisions compared to all other warning messages, including positive messages^76^. In addition, the same cues designed to prevent unhealthy eating behaviour were also demonstrated to lead to altered brain signals associated with self-control when subsequently processing food items^77^. At this stage, it is important to further understand how eating behaviour can be altered to address the obesity epidemic, and our study might inspire research into alternative cueing interventions in the future. However, our findings should also not be over-interpreted, as we neither know the specific drivers for the observed effects nor whether the effects would occur in all possible context situations. Potentially, the results of our study are also a basis for further brain stimulation studies investigating the role of the dlPFC and its connections in obesity. In light of our imaging findings, we show that current intentions of participants (e.g., dieting, stronger desire for immediate gratification) should always be taken into consideration when conducting studies concerning decision-making and cognitive control.

## Electronic supplementary material

Supplementary materials

## Data Availability

The datasets generated and analysed during the current study are available from the corresponding author on reasonable request.

## References

[1] Amlung M, Petker T, Jackson J, Balodis I, MacKillop J (2016). Steep discounting of delayed monetary and food rewards in obesity: a meta-analysis. Psychol Med.

[2] McClelland J (2016). A systematic review of temporal discounting in eating disorders and obesity: Behavioural and neuroimaging findings. Neuroscience & Biobehavioral Reviews.

[3] Vainik U, Dagher A, Dube L, Fellows L (2013). Neurobehavioural correlates of body mass index and eating behaviours in adults: A systematic review. Neuroscience & Biobehavioral Reviews.

[4] Lawyer SR, Boomhower SR, Rasmussen EB (2015). Differential associations between obesity and behavioral measures of impulsivity. Appetite.

[5] Rasmussen EB, Lawyer SR, Reilly W (2010). Percent body fat is related to delay and probability discounting for food in humans. Behavioural Processes.

[6] Weller RE, Cook Iii EW, Avsar KB, Cox JE (2008). Obese women show greater delay discounting than healthy-weight women. Appetite.

[7] Kable JW, Glimcher PW (2010). An “As Soon As Possible” Effect in Human Intertemporal Decision Making: Behavioral Evidence and Neural Mechanisms. Journal of Neurophysiology.

[8] Kable JW, Glimcher PW (2007). The neural correlates of subjective value during intertemporal choice. Nature Neuroscience.

[9] McClure SM, Ericson KM, Laibson DI, Loewenstein G, Cohen JD (2007). Time Discounting for Primary Rewards. Journal of Neuroscience.

[10] McClure SM, Laibson DI, Loewenstein G, Cohen JD (2004). Separate Neural Systems Value Immediate and Delayed Monetary Rewards. Science.

[11] Hare TA, Camerer CF, Rangel A (2009). Self-Control in Decision-Making Involves Modulation of the vmPFC Valuation System. Science.

[12] Hutcherson CA, Plassmann H, Gross JJ, Rangel A (2012). Cognitive Regulation during Decision Making Shifts Behavioral Control between Ventromedial and Dorsolateral Prefrontal Value Systems. The Journal of Neuroscience.

[13] Plassmann H, O’Doherty JP, Rangel A (2010). Appetitive and Aversive Goal Values Are Encoded in the Medial Orbitofrontal Cortex at the Time of Decision Making. The Journal of Neuroscience.

[14] Carter RM, Meyer JR, Huettel SA (2010). Functional neuroimaging of intertemporal choice models: A review. Journal of Neuroscience, Psychology, and Economics.

[15] Lempert, K. M., Steinglass, J. E., Pinto, A., Kable, J. W. & Simpson, H. B. Can delay discounting deliver on the promise of RDoC? *Psychological Medicine*, 1–10, 10.1017/S0033291718001770 (2018). 10.1017/S0033291718001770 30070191

[16] Hare TA, Malmaud J, Rangel A (2011). Focusing Attention on the Health Aspects of Foods Changes Value Signals in vmPFC and Improves Dietary Choice. The Journal of Neuroscience.

[17] Hare, T. A., Hakimi, S. & Rangel, A. Activity in dlPFC and its effective connectivity to vmPFC are associated with temporal discounting. *Frontiers in Neuroscience* **8**, 10.3389/fnins.2014.00050 (2014). 10.3389/fnins.2014.00050 PMC3957025 24672421

[18] Miller EK, Cohen JD (2001). An Integrative Theory of Prefrontal Cortex Function. Annual Review of Neuroscience.

[19] Carter CS, Veen VV (2007). Anterior cingulate cortex and conflict detection: An update of theory and data. Cognitive, Affective, & Behavioral Neuroscience.

[20] Dietrich A, Hollmann M, Mathar D, Villringer A, Horstmann A (2016). Brain regulation of food craving: relationships with weight status and eating behavior. Int J Obes.

[21] Le DSNT (2007). Less activation in the left dorsolateral prefrontal cortex in the reanalysis of the response to a meal in obese than in lean women and its association with successful weight loss. Am J Clin Nutr.

[22] Janssen LK (2017). Loss of lateral prefrontal cortex control in food-directed attention and goal-directed food choice in obesity. NeuroImage.

[23] Volkow ND (2009). Inverse Association Between BMI and Prefrontal Metabolic Activity in Healthy Adults. Obesity.

[24] Kishinevsky FI (2012). fMRI reactivity on a delay discounting task predicts weight gain in obese women. Appetite.

[25] Lempert KM, Phelps EA (2016). The Malleability of Intertemporal Choice. Trends in Cognitive Sciences.

[26] Murawski C (2012). Led into Temptation? Rewarding Brand Logos Bias the Neural Encoding of Incidental Economic Decisions. PLOS ONE.

[27] van der Wal AJ, Schade HM, Krabbendam L, van Vugt M (2013). Do natural landscapes reduce future discounting in humans?. Proceedings of the Royal Society B: Biological Sciences.

[28] Luo S, Ainslie G, Monterosso J (2014). The behavioral and neural effect of emotional primes on intertemporal decisions. Social Cognitive and Affective Neuroscience.

[29] Wilson M, Daly M (2004). Do pretty women inspire men to discount the future? *Proceedings of the Royal Society of London*. Series B: Biological Sciences.

[30] Simmank, J., Murawski, C., Bode, S. & Horstmann, A. Incidental rewarding cues influence economic decisions in people with obesity. *Front Behav Neurosci*, 278, 10.3389/fnbeh.2015.00278 (2015). 10.3389/fnbeh.2015.00278 PMC4606016 26528158

[31] Bode S, Bennett D, Stahl J, Murawski C (2014). Distributed Patterns of Event-Related Potentials Predict Subsequent Ratings of Abstract Stimulus Attributes. PLOS ONE.

[32] Turner WF, Johnston P, de Boer K, Morawetz C, Bode S (2017). Multivariate pattern analysis of event-related potentials predicts the subjective relevance of everyday objects. Conscious Cogn.

[33] Foroni F, Pergola G, Argiris G, Rumiati RI (2013). The FoodCast research image database (FRIDa). Frontiers in Human Neuroscience.

[34] O’Doherty J, Rolls ET, Francis S, Bowtell R, McGlone F (2001). Representation of Pleasant and Aversive Taste in the Human Brain. Journal of Neurophysiology.

[35] Wickham, H. *ggplot2 - Elegant Graphics for Data Analysis* (Springer-Verlag New York, 2009).

[36] RStudio: Integrated Development for R (Boston, MA, 2015).

[37] R: A Language and Environment for Statistical Computing (Vienna, Austria, 2013).

[38] Laibson DG (1997). Eggs and Hyperbolic Discounting. The Quarterly Journal of Economics.

[39] Dietrich A, Federbusch M, Grellmann C, Villringer A, Horstmann A (2014). Body weight status, eating behavior, sensitivity to reward/punishment, and gender: relationships and interdependencies. Front. Psychol..

[40] Horstmann A., Fenske W. K., Hankir M. K. (2015). Argument for a non-linear relationship between severity of human obesity and dopaminergic tone. Obesity Reviews.

[41] Davis C, Fox J (2008). Sensitivity to reward and body mass index (BMI): Evidence for a non-linear relationship. Appetite.

[42] Verdejo-Román J, Vilar-López R, Navas JF, Soriano-Mas C, Verdejo-García A (2017). Brain reward system’s alterations in response to food and monetary stimuli in overweight and obese individuals. Human Brain Mapping.

[43] Brambor T, Clark WR, Golder M (2006). Understanding Interaction Models: Improving Empirical Analyses. Political Analysis.

[44] Smith SM (2002). Fast robust automated brain extraction. Human Brain Mapping.

[45] Jenkinson M, Bannister P, Brady M, Smith S (2002). Improved Optimization for the Robust and Accurate Linear Registration and Motion Correction of Brain Images. NeuroImage.

[46] Pruim RHR (2015). ICA-AROMA: A robust ICA-based strategy for removing motion artifacts from fMRI data. NeuroImage.

[47] Friston KJ (1997). Psychophysiological and Modulatory Interactions in Neuroimaging. NeuroImage.

[48] O’Reilly JX, Woolrich MW, Behrens TEJ, Smith SM, Johansen-Berg H (2012). Tools of the trade: psychophysiological interactions and functional connectivity. Social Cognitive and Affective Neuroscience.

[49] Reimers S, Maylor EA, Stewart N, Chater N (2009). Associations between a one-shot delay discounting measure and age, income, education and real-world impulsive behavior. Personality and Individual Differences.

[50] Amlung M., Petker T., Jackson J., Balodis I., MacKillop J. (2016). Steep discounting of delayed monetary and food rewards in obesity: a meta-analysis. Psychological Medicine.

[51] Bickel WK (2014). Using crowdsourcing to compare temporal, social temporal, and probability discounting among obese and non-obese individuals. Appetite.

[52] Jarmolowicz DP (2014). Robust relation between temporal discounting rates and body mass. Appetite.

[53] Price M, Higgs S, Maw J, Lee M (2016). A dual-process approach to exploring the role of delay discounting in obesity. Physiol Behav.

[54] Heekeren HR, Marrett S, Bandettini PA, Ungerleider LG (2004). A general mechanism for perceptual decision-making in the human brain. Nature.

[55] Heekeren HR, Marrett S, Ruff DA, Bandettini PA, Ungerleider LG (2006). Involvement of human left dorsolateral prefrontal cortex in perceptual decision making is independent of response modality. Proceedings of the National Academy of Sciences of the United States of America.

[56] Bode S (2018). Dissociating neural variability related to stimulus quality and response times in perceptual decision-making. Neuropsychologia.

[57] Shamosh NA (2008). Individual Differences in Delay Discounting Relation to Intelligence, Working Memory, and Anterior Prefrontal Cortex. Psychological Science.

[58] Figner B (2010). Lateral prefrontal cortex and self-control in intertemporal choice. Nature Neuroscience.

[59] Weber BJ, Huettel SA (2008). The neural substrates of probabilistic and intertemporal decision making. Brain Research.

[60] Monterosso JR (2007). Frontoparietal cortical activity of methamphetamine-dependent and comparison subjects performing a delay discounting task. Human Brain Mapping.

[61] Boettiger CA (2007). Immediate Reward Bias in Humans: Fronto-Parietal Networks and a Role for the Catechol-O-Methyltransferase 158Val/Val Genotype. The Journal of Neuroscience.

[62] Cho SS (2010). Continuous theta burst stimulation of right dorsolateral prefrontal cortex induces changes in impulsivity level. Brain Stimul.

[63] Pizzagalli DA, Sherwood RJ, Henriques JB, Davidson RJ (2005). Frontal Brain Asymmetry and Reward Responsiveness A Source-Localization Study. Psychological Science.

[64] Hecht D, Walsh V, Lavidor M (2013). Bi-frontal direct current stimulation affects delay discounting choices. Cognitive Neuroscience.

[65] Weber EU (2007). Asymmetric Discounting in Intertemporal Choice: A Query-Theory Account. Psychological Science.

[66] Fischer GW, Carmon Z, Ariely D, Zauberman G (1999). Goal-Based Construction of Preferences: Task Goals and the Prominence Effect. Manage. Sci..

[67] Weber E, Kirsner B (1997). Reasons for Rank-Dependent Utility Evaluation. Journal of Risk and Uncertainty.

[68] Johnson EJ, Häubl G, Keinan A (2007). Aspects of endowment: A query theory of value construction. Journal of Experimental Psychology: Learning, Memory, and Cognition.

[69] Raichle ME (2001). A default mode of brain function. Proceedings of the National Academy of Sciences.

[70] Chen Z, Guo Y, Feng T (2017). Delay discounting is predicted by scale-free dynamics of default mode network and salience network. Neuroscience.

[71] Kable, J. W. *In Neuroeconomics (Second Edition)* (eds Paul W. Glimcher & Ernst Fehr) 173–192 (Academic Press, 2014).

[72] Woo C-W, Krishnan A, Wager TD (2014). Cluster-extent based thresholding in fMRI analyses: Pitfalls and recommendations. NeuroImage.

[73] Lohmann, G. *et al*. Inflated False Negative Rates Undermine Reproducibility In Task-Based fMRI. *bioRxiv*, 122788, 10.1101/122788 (2017).

[74] Carter CS, Lesh TA, Barch DM (2016). Thresholds, Power, and Sample Sizes in Clinical Neuroimaging. Biological Psychiatry: Cognitive Neuroscience and Neuroimaging.

[75] Kemps E, Tiggemann M, Hollitt S (2014). Exposure to television food advertising primes food-related cognitions and triggers motivation to eat. Psychology & Health.

[76] Rosenblatt Daniel H., Bode Stefan, Dixon Helen, Murawski Carsten, Summerell Patrick, Ng Alyssa, Wakefield Melanie (2018). Health warnings promote healthier dietary decision making: Effects of positive versus negative message framing and graphic versus text-based warnings. Appetite.

[77] Rosenblatt DH (2018). Food product health warnings promote dietary self-control through reductions in neural signals indexing food cue reactivity. Neuroimage Clin.

