# Supplementary material for: Dorsolateral and medial prefrontal cortex mediate the influence of incidental priming on economic decision making in obesity
Source: Sci Rep. 2018 Dec 4;8:17595. doi: 10.1038/s41598-018-35834-1 (PMC6279740; doi:10.1038/s41598-018-35834-1)
Supplement: Supplementary file 1 — Supplementary materials [file 41598_2018_35834_MOESM1_ESM.docx]

Dorsolateral and medial prefrontal cortex mediate the influence of incidental priming on economic decision making in obesity

Filip Morys, Stefan Bode, Annette Horstmann

Supplementary materials and methods:

# Participants

The majority of our participants were registered in the Institute’s database (51 participants) and had previously participated in other studies carried out in the Institute. This database includes a large number of predominantly healthy participants willing to participate in various studies. It predominantly includes students at the University of Leipzig, but also other well-educated individuals of all ages. Unfortunately, the number of individuals with obesity included in the database is comparably low, hence, it was necessary to recruit additional participants with obesity for this study. In total, we recruited 5 new participants, 4 of which were included in the obese sample. These participants were recruited through an online advertisement specific for this study. Given that no participant previously participated in experiments using the same task, and a mixture of more and less experienced participants is common practice in most fields (in particular those requiring samples with specific features), we have no reason to believe that it affected our results in any way. Our sample size was based on a similar behavioural research ^1-3^. However, given that we had to accept some drop-out for our fMRI analysis, which is unfortunately a common problem in studies of this kind, it is possible that there were subtle effects that we might have not been able to detect*.*

Table S1: Demographics table

| Variable | Group | |
| --- | --- | --- |
|  | Lean | Obese |
| Sex | 15 ♀. 15 ♂ | 12 ♀. 14 ♂ |
| BMI [kg/m^2^] | Mean 22.14, SD 1.81, Range 18.43 – 25.48 | Mean 34.32, SD 3.37, Range 29.54 – 44.18 |
| Age [years] | Mean 25.83, SD 3.14, Range 21-38 | Mean 27.42, SD 4.16, Range 19-34 |

# Administration of gustatory stimuli

To administer the gustatory stimuli, three PVC tubes were placed directly in participants’ mouth. The liquid delivery was performed using a computer-controlled, MRI compatible gustometer (Multistimulator OG001, Burghart Messtechnik, Wedel, Germany). The flow rate was set at 500μl/s, which means that during the entire experiment volunteers received 50 ml of liquids.

Table S2. List of pictures used as stimuli in the primed delay discounting task. Pictures were taken from the FoodCast Research Images Database ^4^. The table comprises official database picture names and the content of each image.

| **Positive food pictures** | **Negative food pictures** |
| --- | --- |
| Natural Food 009 - orange | Rotten Food 003 - orange |
| Natural Food 026 - cucumber | Rotten Food 009 - cucumber |
| Natural Food 051 - cheese | Rotten Food 013 - cheese |
| Natural Food 037 - strawberry | Rotten Food 014 - strawberry |
| Natural Food 055 - apple | Rotten Food 018 - apple |
| Natural Food 089 - bread | Rotten Food 025 - bread |


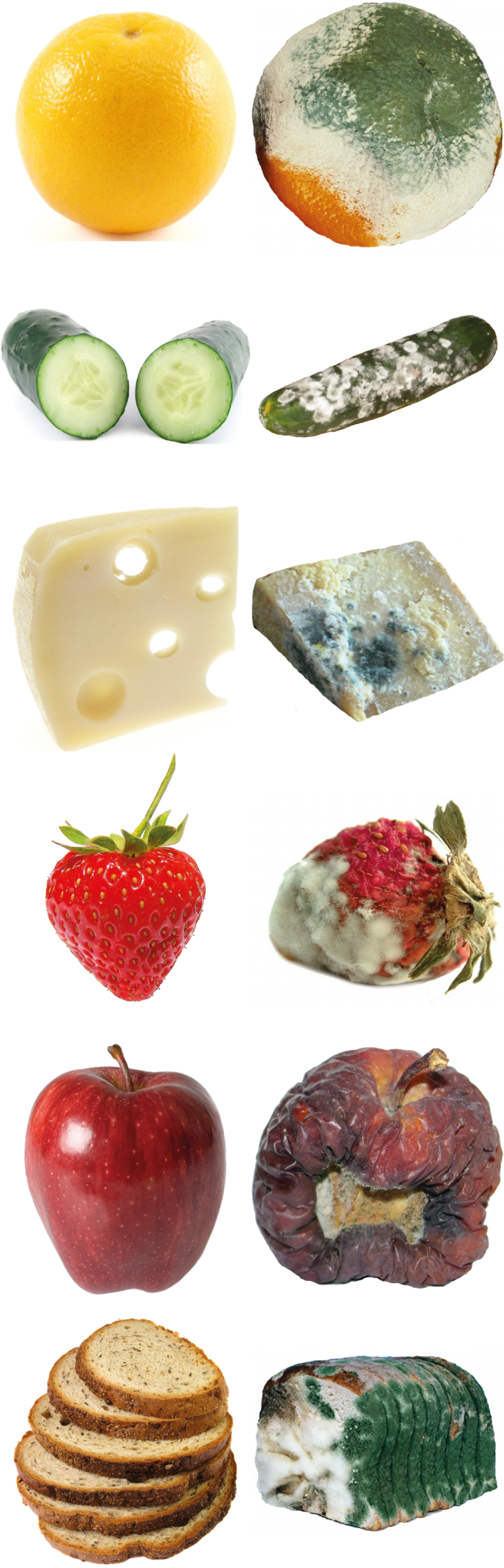


Figure S1. Priming stimuli used in the fMRI task ^4^. The order corresponds to the order in Table S2.

# Questionnaires

After the experiment, participants filled in a set of questionnaires: Beck Depression Inventory (BDI ^5^), Three Factor Eating Questionnaire (TFEQ ^6^), Behavioural Inhibition System and Behavioural Activation System (BIS/BAS ^7^), Zaubermann Time Perception Scale (ZTPS ^8^). We used BDI as a screening tool to potentially exclude participants with depression. The cut-off value was 18 points ^9^ and none of our participants reached it. TFEQ assesses eating behaviour on three dimensions: disinhibition (DI), cognitive restraint (CR) and hunger (H). BIS/BAS captures how a person’s behaviour is driven by reward and punishment. These features often differ between obese and lean participants and may impact on performance in the current task. Additionally, secondary school education, professional qualifications and household income were assessed using a short questionnaire (Table S3).

# Questionnaire data analysis

Questionnaire data were tested for group differences using a two-sample t-test. The income and education assessment questionnaire data were tested for group differences using a chi square test. Moreover, following Simmank et al. ^1^, we tested for within-group correlations between delay discounting factors and subscales of the Three Factor Eating Questionnaire using Spearman’s correlation.

# GLM 1: Perception-related brain activity

We investigated how brain activity changed with respect to the priming stimulation. 14 regressors were entered into the single subject analysis: 12 priming regressors representing the 3 visual and 3 gustatory priming conditions for two trial types (DD and P), and 2 regressors representing the task phase and the response phase (independent of the condition). The priming regressors were time-locked to the onset of each trial, and the duration of each event was set to 500ms (priming length). On the second level, contrasts for individual regressors were entered into a flexible factorial model, with modality and valence as factors. We first calculated an F-contrast to investigate main effects of valence and modality in the brain. Further, to follow up on this, we contrasted gustatory and visual related brain activity against each other (visual > gustatory; visual < gustatory). Moreover, we investigated brain activity related to positive and negative valence (positive > negative; negative < positive; positive > neutral; negative > neutral), independent of modality. In this analysis the F-contrast and results concerning gustatory and visual brain activity were thresholded at an FWE-corrected whole brain level of 0.05 and with an extent cluster threshold of 200 voxels, while all remaining results were thresholded at a whole-brain voxel level p<0.005 and FWE-corrected on a cluster level (p<0.05).

# GLM 2: Choice value- and task-related brain activity

To investigate whether brain activity was modulated by the value of the chosen reward on a trial-by-trial level, we entered the choice value on single subject-level as a parametric modulator of a single regressor containing all DD trials. The model also included a regressor representing task phase for P trials, and two regressors representing priming phase and response phase (independent of conditions). Here (and in the following models) the task regressors were time-locked to the onset of the task screen and the duration of each event was set to 3500ms. We contrasted DD and P trials against each other, as well as investigated where brain activity correlated with choice value. Because of high statistical significance, the results in the DD>P contrast were thresholded at an FWE-corrected whole brain threshold of 0.05 and with an extent cluster threshold of 400 voxels.

# GLM 3: Choice-dependent brain activity

We investigated whether brain activity was modulated by whether participants chose delayed or immediate rewards. To this end, we entered 5 regressors into the single subject analysis: 2 regressors representing DD task phases – one for delayed and one for immediate choices (independent of priming conditions) – 1 regressor representing task phase for P trials, and 2 regressors representing priming and response phases (independent of condition and trial type). Moreover, the first two regressors were parametrically modulated by the choice value on each trial (following the approach described in^10^). Brain activity for immediate choices was compared with brain activity for delayed choices.

Supplementary results:

Table S3. Income and education group differences between lean and obese participants (N=56). Lean and obese groups differed significantly concerning their professional degree with lean participants having a higher professional degree. Total income differed at trend-level between groups with obese individuals having a higher income.

| **Variable** | **Test statistic (Pearson chi-square)** | **Exact p-value** | **Phi coefficient** |
| --- | --- | --- | --- |
| Total income | 14.367 | 0.055 | 0.507 |
| Money available to spend | 7.456 | 0.544 | 0.365 |
| Satisfaction with the income | 4.439 | 0.346 | 0.282 |
| Parents’ income | 9.787 | 0.179 | 0.418 |
| Highest school degree | 4.045 | 0.113 | 0.269 |
| Professional degree | **17.026** | **0.001** | **0.551** |

# Stimuli ratings - between group differences

There were no group differences in evaluation of the gustatory stimuli. Within the visual modality, lean participants rated the negative stimuli as more negative compared to obese participants (main effect for group: F(1,54)=4.817, p=0.032; Table S4).

Table S4. Priming stimuli ratings for lean and obese groups (N=56). Lean participants rated the negative visual stimuli as more negative than the obese participants.

| **Condition** | **Mean** | | **Standard deviation** | | **F value (1,54)** | **p value** | **Effect size \|d\|** |
| --- | --- | --- | --- | --- | --- | --- | --- |
|  | **Lean** | **Obese** | **Lean** | **Obese** |  |  |  |
| Gustatory positive | 76.53 | 79.62 | 22.12 | 20.07 | 0.294 | 0.590 | 0.148 |
| Gustatory neutral | 62.50 | 56.00 | 22.96 | 20.65 | 1.225 | 0.273 | 0.302 |
| Gustatory negative | 19.4 | 16.73 | 21.58 | 16.76 | 0.261 | 0.611 | 0.139 |
| Visual positive | 76.47 | 73.10 | 11.74 | 15.71 | 0.840 | 0.363 | 0.250 |
| Visual neutral | 48.27 | 53.21 | 13.67 | 8.87 | 2.491 | 0.120 | 0.431 |
| Visual negative | 12.82 | 21.31 | 12.08 | 16.76 | **4.817** | **0.032** | **0.599** |

# Stimuli ratings - within modality and valence differences

As expected, significant differences were observed across participants for the rating of the stimuli in relation to both valence and modality. Positive, negative and neutral stimuli were evaluated differently, and visual and gustatory stimuli were evaluated differently (main effects: Modality: F(1,55)=8.372, p=0.005, Valence: F(2,1)=260.003, p<0.001; for further details see Table S5).

Table S5. Ratings for stimuli of different valences and modalities across participants (N=56). Table represents results of a repeated measures ANOVA with modality and valence of stimuli as dependent variables

| **Modality** | **Valence** | **Mean** | **Standard deviation** | **Modality** | | | **Valence** | | |
| --- | --- | --- | --- | --- | --- | --- | --- | --- | --- |
|  |  |  |  | **F value (1,55)** | **p value** | **Partial η^2^** | **F value (2,54)** | **p value** | **Partial η^2^** |
| Gustatory | Positive | 77.96 | 21.06 | **8.372** | **0.005** | **0.132** | **260.003** | **<0.001** | **0.861** |
|  | Neutral | 59.48 | 21.96 |  |  |  |  |  |  |
|  | Negative | 18.16 | 19.36 |  |  |  |  |  |  |
| Visual | Positive | 74.90 | 13.70 |  |  |  |  |  |  |
|  | Neutral | 50.56 | 11.85 |  |  |  |  |  |  |
|  | Negative | 16.76 | 14.93 |  |  |  |  |  |  |

# Sensitivity to reward and punishment, eating behaviour and subjective time perception

We observed significant group differences between lean and obese participants for the disinhibition and cognitive restraint subscales of the TFEQ questionnaire. These results show that obese participants had higher scores on both of those subscales. There were no significant group differences for other questionnaires (cognitive restraint: t(43.195)=-2.864, p=0.006, disinhibition: t(39.688)=-2.191, p=0.034, for further details see Table S6). Further, we did not observe any significant correlations between subscales of the TFEQ and delay discounting parameters (Obese: CR/β: r=0.116, p-0.542; DI/β: r=0.167, p=0.378; H/β: r=0.186, p=0.324; CR/δ: r=0.115, p=0.544, DI/δ: r=0.207, p=0.273, H/δ: r=0.230, p=0.221; Lean: CR/β: r=0.375, p=0.059; DI/β: r=-0.236, p=0.247; H/β: r=-0.188, p=0.358; CR/δ: r=0.039, p=0.849, DI/ δ: r=0.-272, p=0.179, H/δ: r=-0.012, p=0.952;).

Table S6. Group differences in questionnaire outcomes (N=56). Significant differences between groups were observed in the disinhibition and cognitive restraint subscales of the TFEQ.

| **Questionnaire** | **Subscale** | **Mean** | | **Standard deviation** | | **t value** | **p value** | **Effect size \|d\|** |
| --- | --- | --- | --- | --- | --- | --- | --- | --- |
|  |  | **Lean** | **Obese** | **Lean** | **Obese** |  |  |  |
| TFEQ | Cognitive restraint | 5.93 | 8.65 | 2.79 | 4.09 | t(43.195)=**-2.864** | **0.006** | **0.872** |
|  | Disinhibition | 3.93 | 5.62 | 2.05 | 3.42 | t(39.688)=**-2.191** | **0.034** | **0.599** |
|  | Hunger | 5.03 | 4.46 | 2.05 | 3.46 | t(48.081)=0.667 | 0.508 | 0.2 |
| BIS/BAS | BAS drive | 12.07 | 12.08 | 1.68 | 2.26 | t(45.638)=-0.19 | 0.985 | 0.005 |
|  | BAS fun seeking | 11.37 | 12.19 | 1.85 | 2.58 | t(44.599)=-1.359 | 0.181 | 0.365 |
|  | BAS reward responsivity | 15.83 | 16.27 | 1.93 | 2.03 | t(52.005)=-0.819 | 0.416 | 0.222 |
|  | BIS | 19.27 | 18.12 | 2.02 | 2.37 | t(49.408)=1.941 | 0.058 | 0.522 |
| ZTPS | 2 months | 45.77 | 43.96 | 32.06 | 26.73 | t(53.931)=0.230 | 0.819 | 0.061 |
|  | 4 months | 61.87 | 61.85 | 36.26 | 27.33 | t(53.041)=0.002 | 0.998 | 0.001 |
|  | 12 months | 88.20 | 82.50 | 30.28 | 30.48 | t(52.778)=0.700 | 0.487 | 0.188 |

TFEQ – Three Factor Eating Questionnaire, BIS/BAS – Behavioural Inhibition/Behavioural Activation System, ZTPS – Zauberman Time Perception Scale (subjective time perception rated on a scale 0-100 corresponding to ‘not long at all - very long’).

# Baseline delay discounting and group differences

Using a general linear model we investigated whether there were group differences regarding delay discounting parameters for the random choice task. We included weight status and sex as between group factors, β and δ parameters as dependent variables, and obtained professional degree as a covariate (since we found significant group differences in this aspect). Our analysis revealed a significant main effect of weight status on the β parameter (delay-independent bias towards immediate rewards), showing that obese participants had higher delay-independent delay discounting (Figure S2). Moreover, we found a significant weight status by sex interaction for the δ parameter, which is the delay-dependent discount factor (β: weight status main effect: F(1,51)=4.140, p=0.047; δ: weight status * sex interaction: F(1,51)=5.736, p=0.020, for further details see Table S7). This indicates that differences in delay-dependent delay discounting between lean and obese participants were sex-specific (Table S7). Obese females showed higher delay-dependent delay discounting than lean females, while an inverse relationship was observed for males.


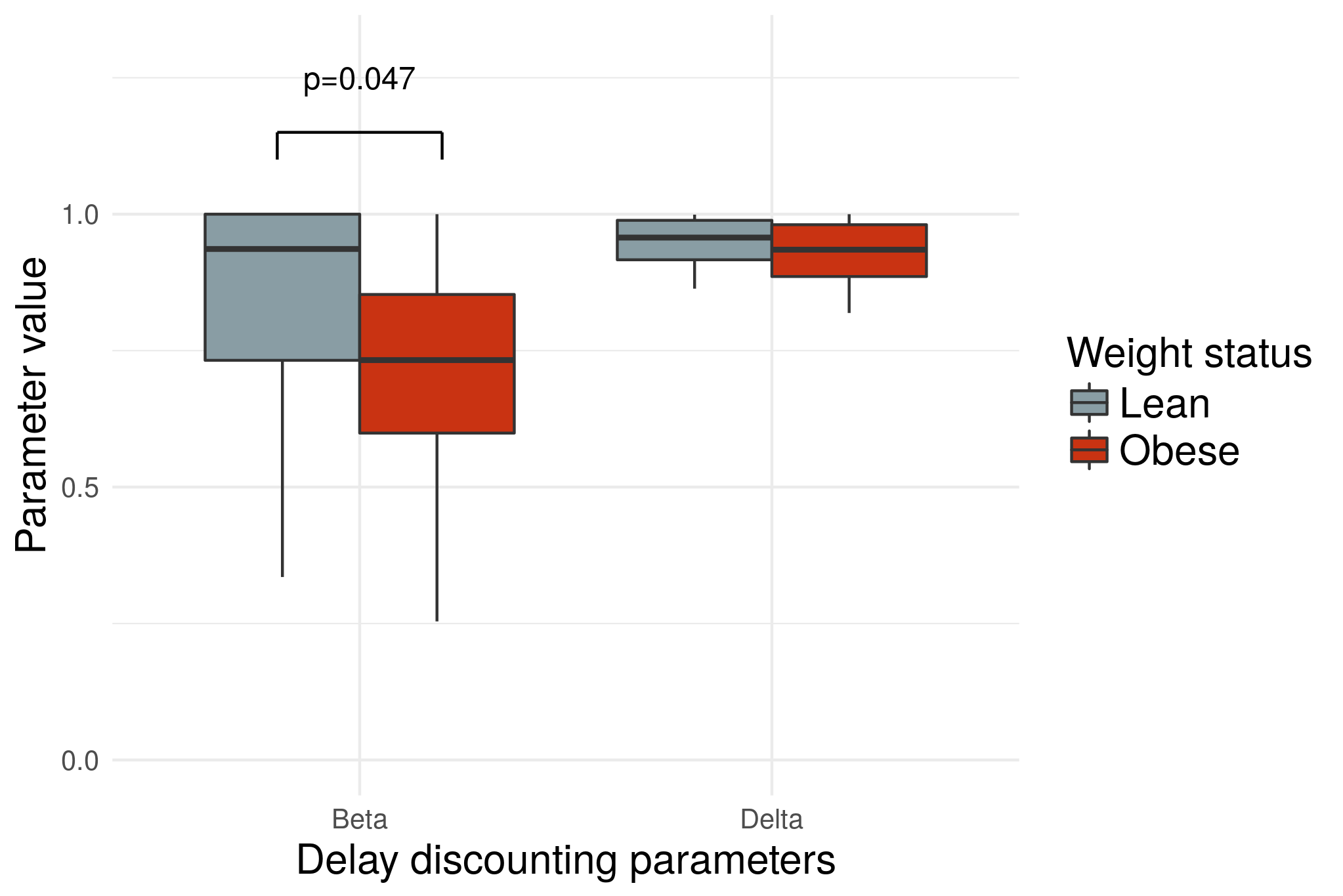


Figure S2. Delay discounting parameters β (delay-independent) and δ (delay-dependent) plotted separately for lean and obese group (N=56). We found a significant group difference between lean and obese participants in the beta parameter of delay discounting representing the present bias (p=0.047). The bold horizontal line represents sample’s median, horizontal lines below and above represent 1^st^ and 3^rd^ quartiles, and the whiskers represent minimum and maximum data points.

Table S7. Group and sex differences for the DD parameters (N=56). This analysis revealed significant group differences for the β (delay-independent) factor for the pooled sample, and β and δ (delay-dependent) parameters differences between obese and lean group for females only. DD – Delay Discounting, β – present bias, δ – discount factor

|  | **Descriptive** | | | | | | **Statistical analysis** | | | | | | | | |
| --- | --- | --- | --- | --- | --- | --- | --- | --- | --- | --- | --- | --- | --- | --- | --- |
| **DD parameter** | **Weight status** | **Mean** | **Standard deviation** | **Sex** | **Mean** | **Standard deviation** | **Weight status** | | | **Sex** | | | **Weight status * sex** | | |
|  |  |  |  |  |  |  | **t value** | **p value** | **Partial η^2^** | **t value** | **p value** | **Partial η^2^** | **t value** | **p value** | **Partial η^2^** |
| **β** | Lean | 0.86 | 0.21 | **Male** | 0.79 | 0.26 | **F(1, 51)=4.140** | **0.047** | **0.075** | F(1,51)= 0.560 | 0.458 | 0.011 | F(1,51)= 2.097 | 0.154 | 0.039 |
|  |  |  |  | **Female** | 0.93 | 0.12 |  |  |  |  |  |  |  |  |  |
|  | Obese | 0.72 | 0.23 | **Male** | 0.74 | 0.29 |  |  |  |  |  |  |  |  |  |
|  |  |  |  | **Female** | 0.70 | 0.14 |  |  |  |  |  |  |  |  |  |
| **δ** | Lean | 0.94 | 0.06 | **Male** | 0.92 | 0.07 | F(1,51)=0.964 | 0.331 | 0.019 | F(1,51)= 0.798 | 0.376 | 0.015 | **F(1,51) = 5.736** | **0.020** | **0.101** |
|  |  |  |  | **Female** | 0.97 | 0.03 |  |  |  |  |  |  |  |  |  |
|  | Obese | 0.93 | 0.05 | **Male** | 0.94 | 0.06 |  |  |  |  |  |  |  |  |  |
|  |  |  |  | **Female** | 0.92 | 0.04 |  |  |  |  |  |  |  |  |  |

# Brain activity related to perceptual processing

To assess differential perceptual brain activity we first calculated an F-contrast over all conditions. This resulted in three large clusters covering almost the entire grey matter of the brain. To follow-up on this finding, we contrasted gustatory stimulation versus visual stimulation. For the gustatory stimulation > visual stimulation contrast, we observed higher activity in five clusters: the bilateral Rolandic operculum and insula, the supplementary motor area, and the superior frontal and cingulate gyri (Figure S3). The visual stimulation > gustatory stimulation contrast elicited higher brain activity in three clusters over the bilateral fusiform gyri and calcarine fissure (Figure S4).

We then contrasted negative and positive stimulation, regardless of modality, versus neutral stimulation. We found that negative stimulation elicited higher activation in four distinct clusters, including the superior and middle frontal gyri, thalamus, amygdala, insula, cingulate gyrus and the bilateral inferior occipital gyri. Positive stimulation elicited higher brain activation in five distinct clusters, including the bilateral inferior occipital gyrus, bilateral insula and amygdala, bilateral inferior orbital frontal gyrus, and the midbrain, including the ventral tegmental area. Clusters surviving a more stringent voxel-wise threshold of 0.001 are denoted in Table S8. Additionally, we directly contrasted positive and negative stimulation-related brain activity against each other. This analysis revealed that brain activity in the postcentral gyrus and left dorsolateral prefrontal and orbitofrontal cortex was higher for negative than for positive stimulation. We found no significant brain activations for the opposite contrast (Table S8).

# Choice value-related brain activity

This analysis aimed at identifying brain regions, which tracked monetary values chosen in the DD task. For this, we investigated how brain activity was parametrically modulated by this chosen value on a trial-by-trial basis. In line with previous findings, the value of the chosen reward was reflected in brain activity in a single cluster with its maximum in the left medial frontal gyrus (Table S9, figure S5).

# Task-related brain activity

We investigated which brain regions were activated during performance of the delay discounting task in general, irrespective of the priming conditions. To control for possible perceptual confounds related to priming stimulation, we contrasted DD trials with P trials. We found a number of significant clusters, including the bilateral visual cortex, left middle and superior frontal gyri, right precentral gyrus, and right superior parietal lobule. Further, we investigated whether brain activity differed for immediate and delayed choices. We identified significant clusters for the immediate > delayed choices contrast only. These included the bilateral visual cortices along with parts of the cerebellum, the right middle frontal gyrus, and the precuneus (Table 10). We did not find significant clusters for the delayed > immediate contrast. Clusters surviving a more stringent voxel-wise threshold of 0.001 are denoted in Table 10.


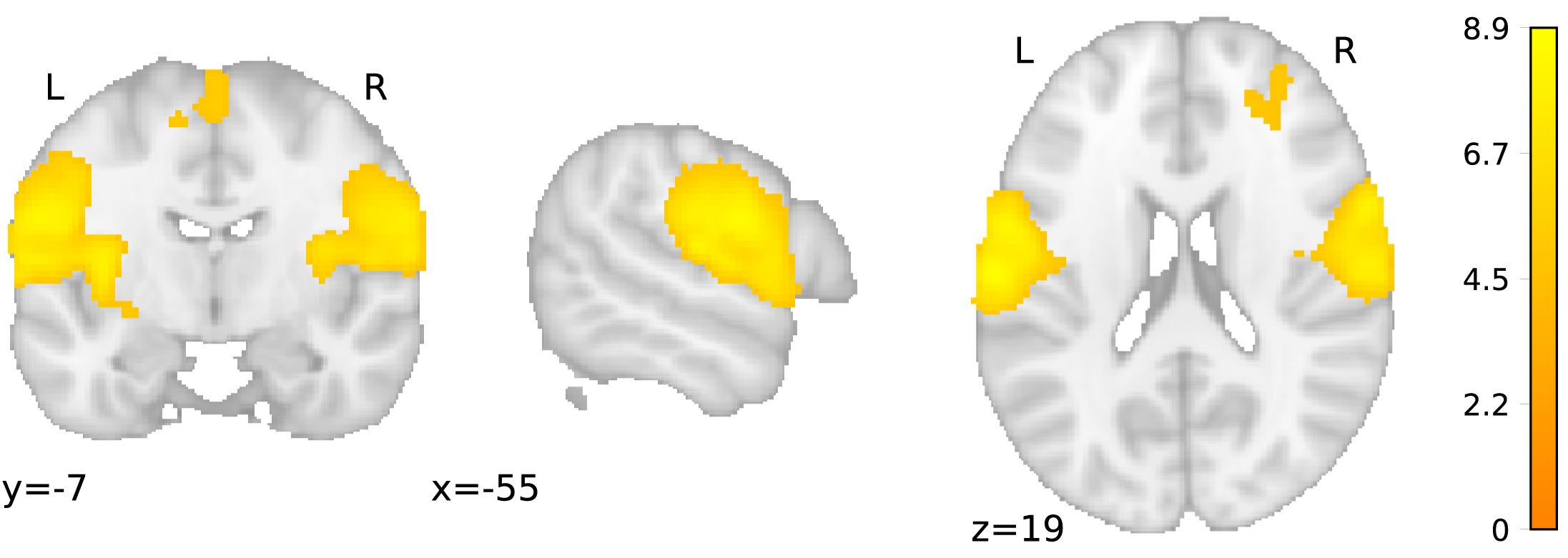


Figure S3. Brain regions showing higher activity for trials using gustatory stimulation (priming phase, independent of DD or P trial type) compared to trials using visual stimulation. L – left, R – right. T-values are plotted on a standard brain (N=51, whole brain p_FWE_=0.007).


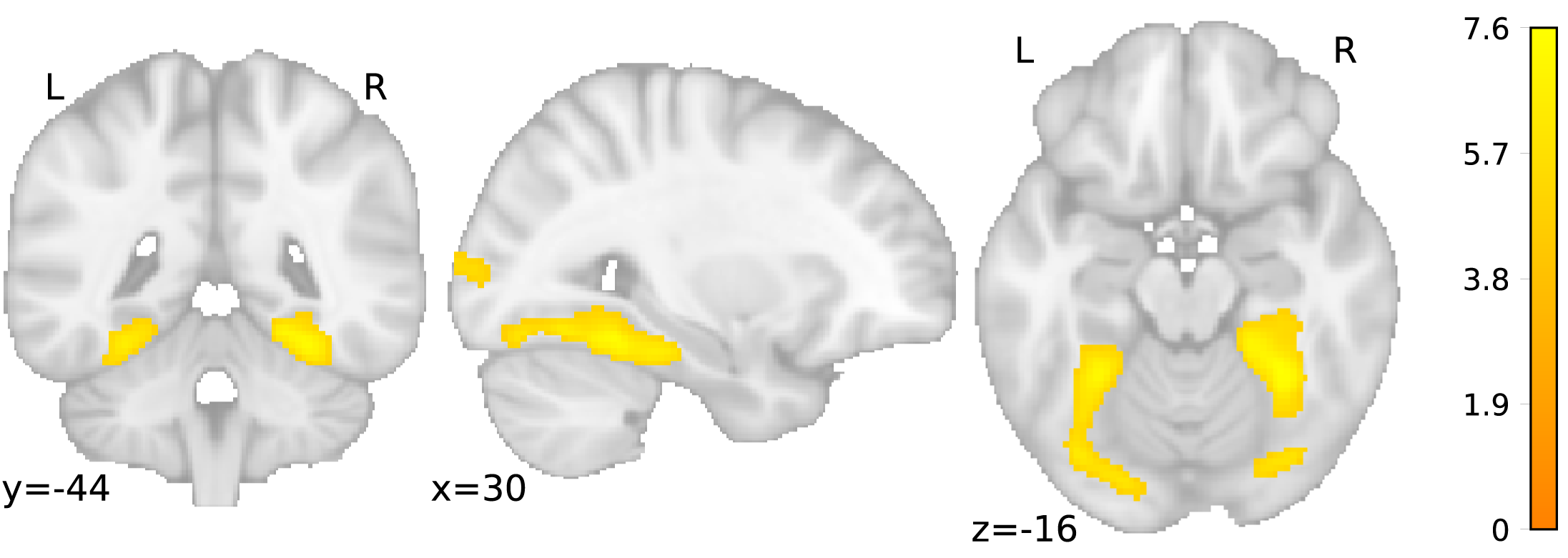


Figure S4. Brain regions showing higher activity for trials using visual stimulation (priming phase, independent of DD or P trial type) compared to trials using gustatory stimulation. L – left, R – right. T-values are plotted on a standard brain (N=51, whole brain p_FWE_=0.05).


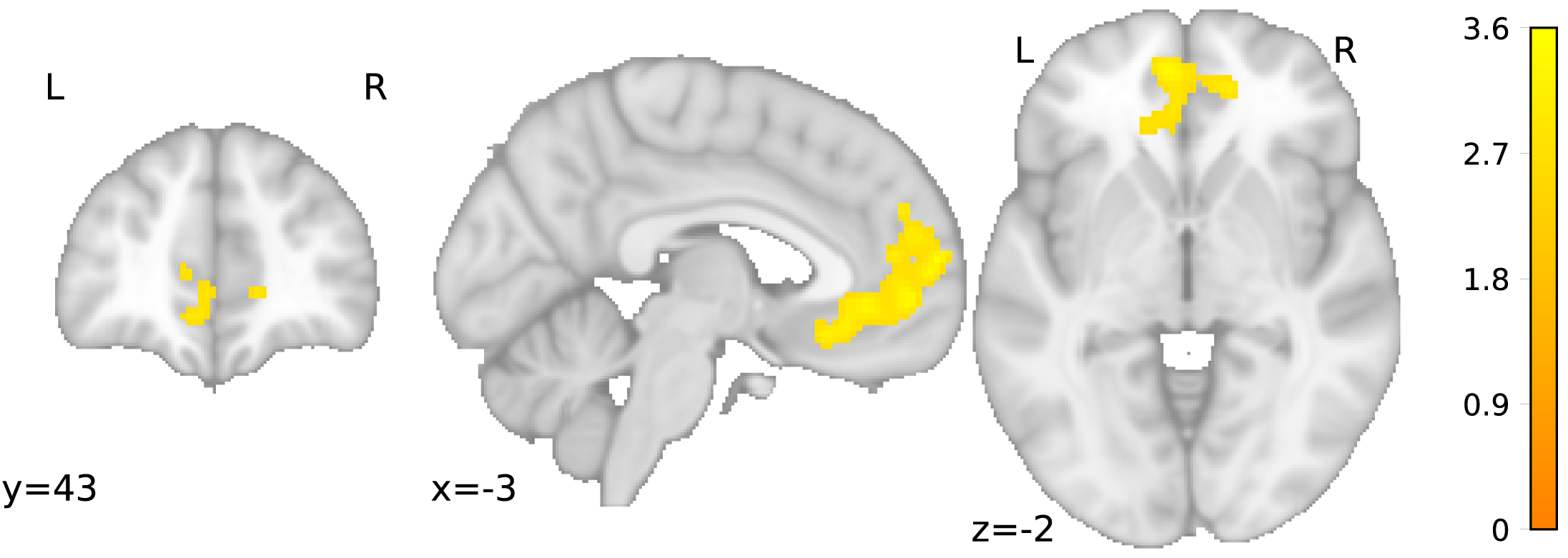


Figure S5. Brain regions where the trial-wise choice value parametrically modulated brain activity during DD trials. L – left, R – right. T-values are plotted on a standard brain (N=51 cluster defining threshold 0.005, p_FWE_=0.007).

Table S8. Brain regions showing significant effects for the perceptual analysis (N=51).

| **Contrast** | **Region of the peak voxel** | **Cluster size [voxels]** | **Coordinates (MNI)** | | | **Peak z score** | **Peak t score** |
| --- | --- | --- | --- | --- | --- | --- | --- |
| F-contrast** | Middle occipital gyrus L | 123613 | -26 | -92 | 4 | - | F: 8260.21 |
|  | Inferior orbital frontal gyrus R | 607 | 30 | 38 | -20 | - | F: 355.12 |
|  | Cingulum R | 520 | 2 | -32 | 26 | - | F: 165.77 |
| Gustatory>Visual** | Postcentral gyrus L | 4443 | -62 | -16 | 16 | - | 8.92 |
|  | Precentral gyrus R | 3260 | 60 | 4 | 24 | 7.67 | 8.16 |
|  | Medial frontal gyrus R | 1457 | 2 | -2 | 60 | 6.15 | 6.40 |
|  | Cingulate gyrus R | 568 | 6 | 12 | 36 | 5.65 | 5.84 |
|  | Superior frontal gyrus R | 265 | 28 | 44 | 12 | 5.32 | 5.47 |
| Visual>Gustatory** | Fusiform gyrus R | 1046 | 34 | -50 | -18 | 7.18 | 7.57 |
|  | Calcarine Fissure R | 626 | 18 | -98 | 2 | 6.86 | 7.20 |
|  | Fusiform gyrus L | 746 | -30 | -50 | -16 | 6.74 | 7.07 |
| Negative>Neutral | Insula L* | 25940 | -36 | 8 | -10 | 6.96 | 7.32 |
|  | Middle occipital gyrus L* | 1730 | -48 | -82 | -12 | 6.73 | 7.05 |
|  | Inferior occipital gyrus R* | 987 | 50 | -80 | -12 | 5.52 | 5.69 |
|  | Precuneus* | 931 | 0 | -74 | 40 | 4.21 | 4.29 |
| Positive>Neutral | Middle occipital gyrus L* | 2922 | -48 | -82 | -12 | 7.28 | 7.69 |
|  | Insula L* | 3779 | -36 | 6 | -10 | 6.64 | 6.95 |
|  | Insula R* | 2805 | 38 | 4 | -10 | 6.02 | 6.25 |
|  | Middle occipital gyrus R* | 2208 | 52 | -78 | -12 | 5.87 | 6.08 |
|  | Superior frontal gyrus R | 832 | 16 | 10 | 72 | 3.79 | 3.84 |
| Negative>Positive | Middle frontal gyrus L | 3616 | -36 | 56 | 2 | 4.59 | 4.13 |

R – right, L – left; * denotes clusters surviving 0.001 voxel-wise threshold and further FWE correction (0.007); ** this analysis was performed using an FWE-corrected voxel-wise threshold of 0.05

Table S9. Brain region modulated by the single trial monetary choice value on the delay discounting task (N=51).

| **Contrast** | **Region of the peak voxel** | **Cluster size [voxels]** | **Coordinates (MNI)** | | | **Peak z score** | **Peak t score** |
| --- | --- | --- | --- | --- | --- | --- | --- |
| Parametric modulation choice value | Medial frontal gyrus L | 1056 | -4 | 62 | 10 | 3.37 | 3.61 |

R – right, L - left

Table S10. Brain regions showing higher activity for the delay discounting task conditions vs. perception only conditions and for the immediate vs. delayed choices (N=36).

| **Contrast** | **Region of the peak voxel** | **Cluster size [voxels]** | **Coordinates (MNI)** | | | **Peak z score** | **Peak t score** |
| --- | --- | --- | --- | --- | --- | --- | --- |
| DD>P** | Calcarine fissure L | 8108 | -16 | -96 | -6 | - | 12.42 |
|  | Middle occipital gyrus R | 2017 | 32 | -98 | -4 | - | 12.38 |
|  | Medial frontal gyrus L | 2321 | -6 | -2 | 58 | 6.21 | 7.78 |
|  | Precentral gyrus R | 1573 | 30 | -24 | 62 | 6.21 | 7.78 |
|  | Inferior frontal gyrus L | 809 | -46 | 10 | 30 | 5.83 | 7.11 |
|  | Precuneus L | 815 | -26 | -64 | 38 | 5.65 | 6.81 |
|  | Superior parietal lobule R | 615 | 34 | -54 | 50 | 5.48 | 6.53 |
| Immediate choices > Delayed choices | Middle frontal gyrus R* | 4889 | 26 | 70 | 10 | 5.01 | 6.25 |
|  | Cerebellum R* | 12183 | 2 | -86 | -20 | 4.54 | 5.43 |
|  | Cingulate Gyrus L | 1200 | -6 | -8 | 28 | 3.97 | 4.56 |
|  | Medial frontal gyrus R | 1079 | 6 | 18 | 48 | 3.89 | 4.44 |

DD – delay discounting, P – perception only, L – left, R – right; * denotes clusters surviving 0.001 voxel-wise threshold and further FWE correction (0.007); ** this analysis was performed using an FWE-corrected voxel-wise threshold of 0.05

**The following 7 figures depict scatter plots of relationships of BMI with other measures for all analyses where BMI was included as a continuous predictor:**


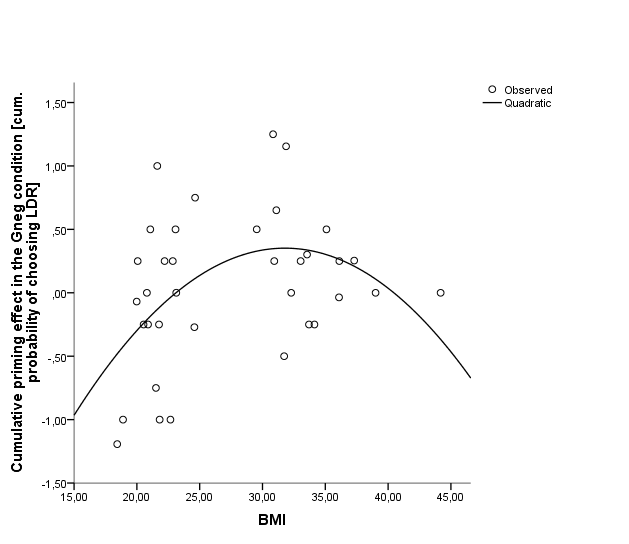


Figure S6. Scatter plot of the relationship between BMI and the cumulative priming effect in the gustatory negative condition (R^2^=0.208, p=0.021, for details see section 3.4.)


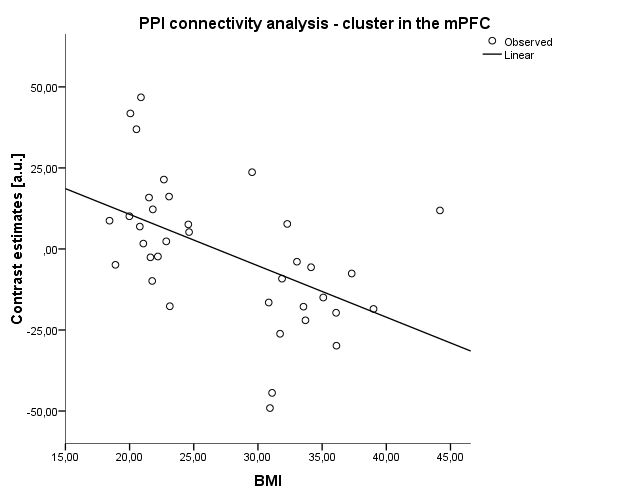


Figure S7. Scatter plot of the relationship between BMI and the PPI connectivity (GLM 6) to the medial prefrontal cortex (mPFC) – Figure presented for illustration purposes; for details see section 3.6.6; a.u. – arbitrary units


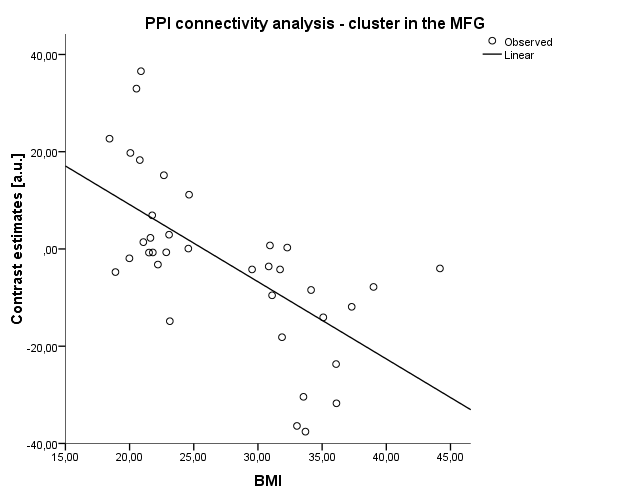


Figure S8. Scatter plot of the relationship between BMI and the PPI connectivity (GLM 6) to the middle frontal gyrus (MFG) – Figure presented for illustration purposes; for details see section 3.6.6; a.u. – arbitrary units


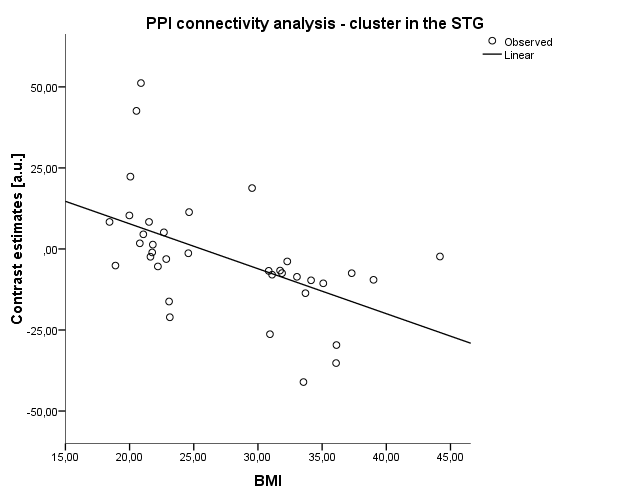


Figure S9. Scatter plot of the relationship between BMI and the PPI connectivity (GLM 6) to the superior temporal gyrus (STG) – Figure presented for illustration purposes; for details see section 3.6.6; a.u. – arbitrary units


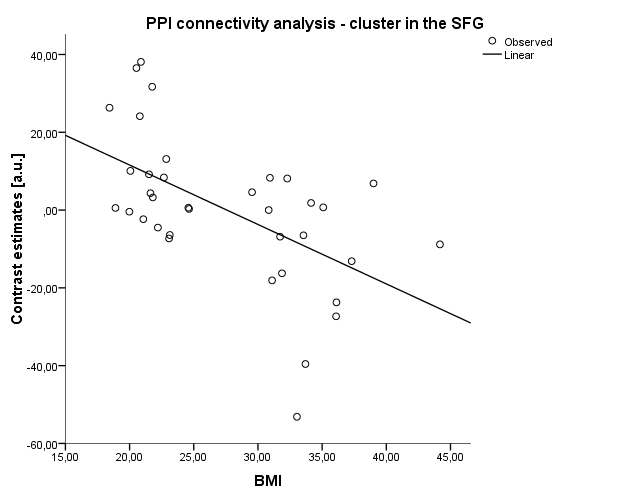


Figure S10. Scatter plot of the relationship between BMI and the PPI connectivity (GLM 6) to the superior frontal gyrus (SFG) – Figure presented for illustration purposes; for details see section 3.6.6; a.u. – arbitrary units


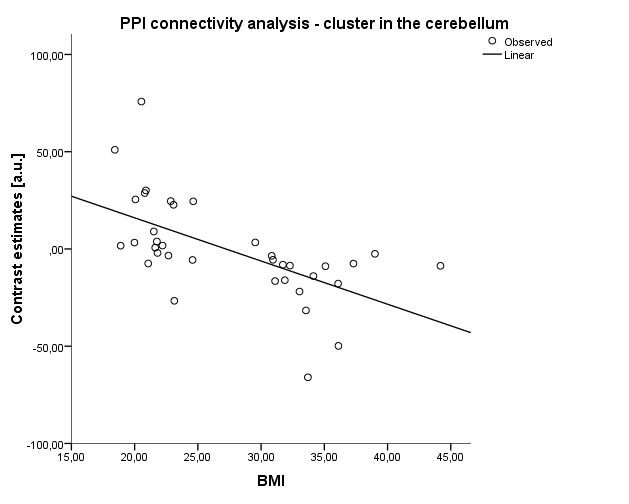


Figure S11. Scatter plot of the relationship between BMI and the PPI connectivity (GLM 6) to the cerebellum – Figure presented for illustration purposes; for details see section 3.6.6; a.u. – arbitrary units


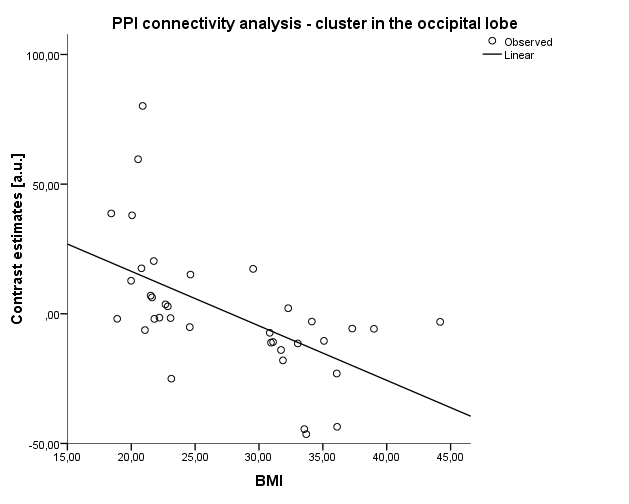


Figure S12. Scatter plot of the relationship between BMI and the PPI connectivity (GLM 6) to the occipital lobe – Figure presented for illustration purposes; for details see section 3.6.6; a.u. – arbitrary units

**
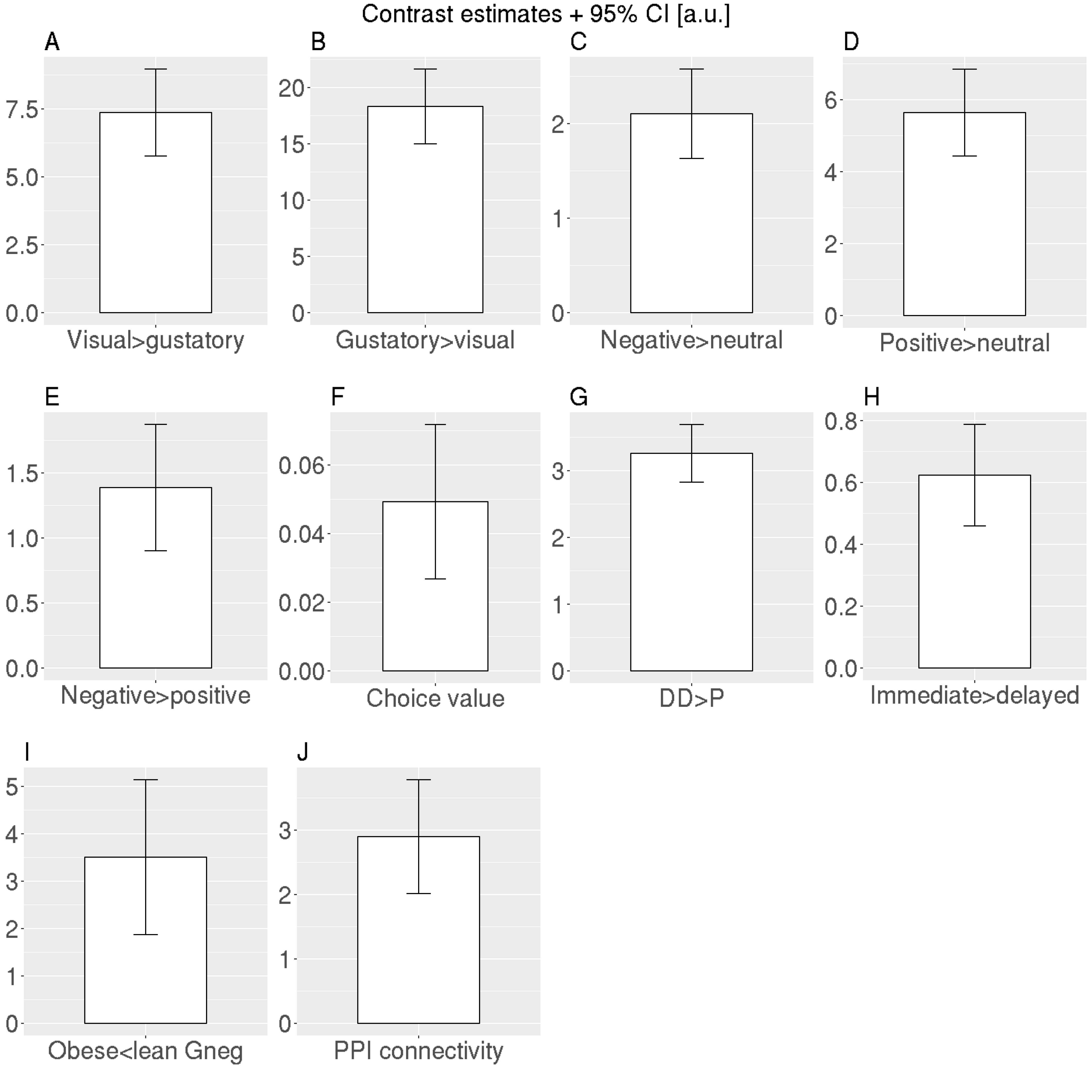
**

Figure S13. Figure depicting contrast estimates and 95% confidence intervals (CI) for peak voxels of each of the fMRI analyses (see Tables S8-S10 and 3-4). **A** analysis contrasting gustatory and visual stimulation; **B** analysis contrasting visual and gustatory stimulation; **C** analysis contrasting negative and neutral stimulation; **D** analysis contrasting positive and neutral stimulation; **E** analysis contrasting negative and positive stimulation; **F** analysis investigating modulation of brain activity by choice value during the delay discounting task; **G** analysis contrasting delay discounting trials with perception only trials; **H** analysis contrasting immediate choices with delayed choices on delay discounting trials; **I** analysis contrasting brain activity during the gustatory negative condition in the obese and in the lean group; **J** PPI analysis. Please note different y-axis scales for each subplot.

Supplementary references:

1 Simmank, J., Murawski, C., Bode, S. & Horstmann, A. Incidental rewarding cues influence economic decisions in people with obesity. *Front Behav Neurosci*, 278, doi:10.3389/fnbeh.2015.00278 (2015).

2 Murawski, C., Harris, P. G., Bode, S., Domínguez D, J. F. & Egan, G. F. Led into Temptation? Rewarding Brand Logos Bias the Neural Encoding of Incidental Economic Decisions. *PLOS ONE* **7**, e34155, doi:10.1371/journal.pone.0034155 (2012).

3 Luo, S., Ainslie, G. & Monterosso, J. The behavioral and neural effect of emotional primes on intertemporal decisions. *Social Cognitive and Affective Neuroscience* **9**, 283-291, doi:10.1093/scan/nss132 (2014).

4 Foroni, F., Pergola, G., Argiris, G. & Rumiati, R. I. The FoodCast research image database (FRIDa). *Frontiers in Human Neuroscience* **7**, 51, doi:10.3389/fnhum.2013.00051 (2013).

5 Beck, A. T., Steer, R. A., Ball, R. & Ranieri, W. F. Comparison of Beck Depression Inventories-IA and-II in Psychiatric Outpatients. *Journal of Personality Assessment* **67**, 588-597, doi:10.1207/s15327752jpa6703_13 (1996).

6 Stunkard, A. J. & Messick, S. The three-factor eating questionnaire to measure dietary restraint, disinhibition and hunger. *Journal of Psychosomatic Research* **29**, 71-83, doi:10.1016/0022-3999(85)90010-8 (1985).

7 Carver, C. S. & White, T. L. Behavioral inhibition, behavioral activation, and affective responses to impending reward and punishment: The BIS/BAS Scales. *Journal of Personality and Social Psychology* **67**, 319-333, doi:10.1037/0022-3514.67.2.319 (1994).

8 Zauberman, G., Kim, B. K., Malkoc, S. A. & Bettman, J. R. Discounting Time and Time Discounting: Subjective Time Perception and Intertemporal Preferences. *Journal of Marketing Research* **46**, 543-556, doi:10.1509/jmkr.46.4.543 (2009).

9 Hautzinger, M. *BDI: Beck-Depressions-Inventar ; Testhandbuch*. 2., überarb. Aufl edn, (Huber, 2006).

10 Hare, T. A., Hakimi, S. & Rangel, A. Activity in dlPFC and its effective connectivity to vmPFC are associated with temporal discounting. *Frontiers in Neuroscience* **8**, doi:10.3389/fnins.2014.00050 (2014).
